# Bacteria responsive polyoxometalates nanocluster strategy to regulate biofilm microenvironments for enhanced synergetic antibiofilm activity and wound healing

**DOI:** 10.7150/thno.49008

**Published:** 2020-08-08

**Authors:** Yuetong Zhang, Yang Pi, Yusheng Hua, Jiani Xie, Chengyan Wang, Kun Guo, Zhigang Zhao, Yuan Yong

**Affiliations:** 1 College of Chemistry and Environment Protection Engineering, Southwest Minzu University, Chengdu 610041, China.; 2 CAS Key Laboratory for Biomedical Effects of Nanomaterials and Nanosafety, Institute of High Energy Physics, Chinese Academy of Sciences, Beijing100040, China.; 3 College of Pharmacy, Southwest Minzu University, Chengdu 610041, China.

**Keywords:** Biofilm, polyoxometalate, acid/reductive-responsive, peroxidase-like activity, photothermal antibacterial effect

## Abstract

**Backgroud:** Nowadays, biofilms that are generated as a result of antibiotic abuse cause serious threats to global public health. Such films are the primary factor that contributes to the failure of antimicrobial treatment. This is due to the fact that the films prevent antibiotic infiltration, escape from innate immune attacks by phagocytes and consequently generate bacterial resistance. Therefore, exploiting novel antibacterial agents or strategies is extremely urgent.

**Methods:** Herein, we report a rational construction of a novel biofilm microenvironment (BME)-responsive antibacterial platform that is based on tungsten (W)-polyoxometalate clusters (POMs) to achieve efficient bactericidal effects.

**Results:** On one hand, the acidity and reducibility of a BME could lead to the self-assembly of POMs to produce large aggregates, which favor biofilm accumulation and enhance photothermal conversion under near-infrared (NIR) light irradiation. On the other hand, reduced POM aggregates with BME-induced photothermal-enhanced efficiency also exhibit surprisingly high peroxidase-like activity in the catalysis of bacterial endogenous hydrogen peroxide (H_2_O_2_) to produce abundant reactive oxygen species (ROS). This enhances biofilm elimination and favors antibacterial effects. Most importantly, reduced POMs exhibit the optimal peroxidase-like activity in an acidic BME.

**Conclusion:** Therefore, in addition to providing a prospective antibacterial agent, intelligent acid/reductive dual-responsive POMs will establish a new representative paradigm for the areas of healthcare with minimal side effects.

## Introduction

Bacterial infection diseases remain a major public health concerns to worldwide because of the high morbidity and mortality. 1-5. In particular, the efficiency of bacteria that are disposed of via conventional antibiotics has been threatened by the developing emergence of antibiotic resistance. This is mainly because of structure changes and genetic mutations of planktonic bacteria and the formation of biofilms 6, 7. Biofilms are self-generated protective communities on an extracellular matrix that are composed of healthy bacterial cells. Biofilms irreversibly adhere to the surface of a sample or organ and can be embedded in an extracellular polymeric substance (EPS) matrix, which is mainly composed of extracellular bacterial DNA, exopolysaccharides, proteins and enzymes 7, 8. As the major component of biofilms, an EPS protects pathogenic bacteria from attack by extraneous antibiotics. This is attributed to lowered biofilm penetration, which consequently induces bacterial resistance to antibacterial agents and causes antibiotic treatments to fail 9-13. Additionally, because the encapsulation of EPS generates highly acidic niches (pH values close to 4.5), a biofilm microenvironment (BME) that is infected with bacteria is characterized as hypoxic and more acidic than the microenvironment of healthy tissues, and this leads to failure to eradicate infectious disease and to the recurrence of infection 7, 14, 15. Thus, novel anti-biofilm strategies that target bacterial-infected microenvironments are important and have attracted significant interest. Specially, they have the potential to open new treatment paradigms for infections.

Recently, numerous strategies that take advantage of a unique bacterial-infected microenvironment as an important regulated target to facilitate the destruction of biofilm structure and to degrade the EPS have attracted widespread attention 16-18. These strategies include chemical-mediated approaches using antibiotics, antibacterial peptides, quaternary ammonium compounds, and peptidepolysaccharides 19, 20, nonchemotherapeutic means such as nanotechnology-based strategies that involve the unique antibacterial nanostructures themselves and external physical stimuli such as light, electricity, magnetism and heat-activated alternative therapies 21-37. Among these therapies, nanoparticle-based antibacterial agents have recently demonstrated their potential in the suppression of biofilm formation. This is achieved via reducing bacterial adhesion and viability, as well as regulating the unique bacterial-infected microenvironment as a method of inducing transformation. These new strategies can enhance antibacterial properties without causing side-effects and are able to overcome drug-resistance mechanisms with a high, sustained locally administrated dosage. Therefore, a promising strategy that is based on a combination of antibacterial agents is highly desirable for combating bacterial infectious disease. This is probably beneficial for regulating bacterial-infected microenvironments, overcoming biofilm recalcitrance, reducing antibiotic resistance and increasing anti-biofilm activity.

Among several antibacterial agents, hydrogen peroxide (H_2_O_2_), which is an endogenously active molecule, plays a critical role in physiological and pathophysiological processes such as wound disinfection. However, the concentration of H_2_O_2_ that is traditionally used is in the range of 166 mM to 1 M (volume ratio: 0.5-3 %) 38-40, and this prevents wound healing and damages adjacent normal organs during bacterial sterilization. Currently, various novel types of peroxidase-like nanomaterials that have high peroxidase-mimic activity, such as carbon-based nanomaterials 41-44, noble metals 45-51, iron-based nanomaterials 52-55, transitional metal dichalcogenides 56, 57, and polyoxometalates 58, have been known to have obvious antibacterial effects. They can disrupt the integrity of bacterial cell membranes and cell wall permeability. In particular, they restrain bacterial adhesion and biofilm formation. Unfortunately, these peroxidase-like nanomaterials that have high peroxidase-mimic activity still have disadvantageous, such as their intrinsic cytotoxicity, and because they generate oxidative stress at low concentrations of H_2_O_2_, and increase antimicrobial resistance under single antibacterial methods. In this regard, the development of new biocompatible and intelligent peroxidase-like nanostructures with BME stimuli-responsive character and the performance of the combination of multiple antibacterial substances are highly desirable for enhancing antibacterial efficiency and for achieving better antibacterial outcomes with potential therapeutic effect and decreased inevitable side-effects.

Herein, we report a representative acid/reductive-responsive inorganic paradigm as a result of tungsten (W)-based POM clusters (GdW_10_O_36_ NCs) for the regulation of BMEs to achieve efficient bactericidal effects and wound healing. Owing to the unique self-adaptive electronic structure and chemical versatility of POMs, such as polyanionic feature, rigid framework, monodispersed size, versatile properties, and biofunctionality, this representative W-based POM can self-assemble into larger aggregates in bacteria under the activation by the BME via protonation-triggered hydrogen bonding formation. This promotes effective adhesion to bacteria that have negatively charged cell surfaces within biofilms. Meanwhile, a reductive BME can “regenerate” the strong NIR absorption of POM through the chemical conversion of GSH-promoted W (VI) to W (V), which significantly increases their photothermal conversion performance. Most importantly, the obtained reduced POM aggregates in the BME have self-adaptive enhanced photothermal conversion, they catalyze endogenous hydrogen peroxide (H_2_O_2_) to generate abundant hydroxide radicals (•OH) via intrinsic peroxidase-like catalytic activity. This increases the obliteration of biofilm and cause bacteria to be more vulnerable to damage; they also can strengthen the antibacterial effect via the combination of NIR laser-induced hyperthermia with a low endogenous concentration of H_2_O_2_. Therefore, we believe that this work will advance the development of these acid/reductive-responsive POMs in the field of enzymatic catalysis and areas of healthcare.

## Methods

### Materials

All of the reagents and solvents were used as received without any modification. Sodium tungstate dehydrate (Na_2_WO_4_·2H_2_O), gadolinium chloride hexahydrate (GdCl_6_·6H_2_O), glutathione (GSH), and L-ascorbic acid (AA) were obtained from Alfa Aesar Reagent Company (China, Shanghai). Tetramethyl benzidine (TMB), o-phenylenediamine (OPD), 2,2'-azino-bis(3-ethylbenzothiazoline-6-sulfonic acid) diammonium salt (ABTS), 3, 3'-diaminobenzidine (DAB), horseradish peroxidase (HRP), propidium iodide (PI) and calcein (AM) were all obtained from Aladdin Company (China, Shanghai). P-Phthalic acid (TA) was obtained from the Chengdu area of the industrial development zone Xindu Mulan. Crystal violet staining solution was obtained from Scientific Phygene. All of the other chemical agents were used as received without any modification.

### Preparation of GdW_10_O_36_ nanoclusters before and after reduction

Na_9_[Gd(W_5_O_18_)_2_]·×H_2_O nanoclusters (GdW_10_O_36_ NCs) were fabricated via a facile recrystallization method that has previously been described 59, 60. Briefly, 25 mmol of Na_2_WO_4_·2H_2_O was dispersed in 20 mL of ultrapure water under continuous stirring at room temperature to form a homogeneous solution. A solution of acetic acid was then added dropwise until the pH was in the range of 7.40-7.50. Subsequently, 1.25 mmol/mL GdCl_6_·6H_2_O (477.95 g mol^-1^, 2.0 mL) aqueous solution was added dropwise to this system under continuous stirring and heated to 85 °C until a transparent solution formed. Finally, crude crystals were isolated from solution when the mixtures were cooled to room temperature. To obtain the reduced GdW_10_O_36_ nanoclusters (reduced GdW_10_O_36_ NCs), another solution of glutathione with a different concentration or a solution of glycol was injected dropwise into the system, which was irradiated with a hand-held ultraviolet lamp. After irradiation for 12 h, water and ethanol were added three times to precipitate the reduced clusters, which were then dried in a freeze dryer to obtain reduced cluster powders.

### Peroxidase-like catalytic activity of reduced GdW_10_O_36_ nanoclusters

TMB (1 mM) and OPD (1.85 mM) were chosen to evaluate the capacity of reduced GdW_10_O_36_ NCs (33 μg/mL) dispersed in tris buffer solution containing Na_2_HPO_4_·12H_2_O (0.2 M) and citric acid (0.1 M) to convert H_2_O_2_ (33 mM) into •OH radicals in the absence or presence of 808 nm NIR laser irradiation for 30 min. UV-Vis spectrophotometer (UV-Vis, UV-6100, MAPADA) was used to monitor the absorbance change of the characteristic peak at 652 nm or 450 nm. In addition, TA was used as a probe to react with the generated •OH to form TAOH, which is highly fluorescence. Fluorescence spectroscopy (HORIBA Dual-FL) was then used to record fluorescence spectra of samples after different treatments. Also the color change of peroxidase-like reduced GdW_10_O_36_ NCs was also photographed after 30 min of co-incubation at room temperature.

### Bacterial culture and dose-dependent antibacterial ability of reduced GdW_10_O_36_ nanoclusters

*E.coli* (ATCC 25922) and *S.aureus* (ATCC 29213) bacteria were planted in a Luria-Bertani (LB) agar plate and then moved to 10 mL of LB with shaking for 8 h at 37 °C. When the logarithmic phase (A600 = 0.4) was achieved, Bacteria were collected via centrifugation at a speed of 8000 rpm for 1 min. The bacteria that collected at the bottom were washed and resuspended in phosphate buffered solution (PBS, 10 mM, pH = 7.4), which was attenuated to ~ 10^6^ colony-forming units (CFU∙mL^-1^) for subsequent experiments. To evaluate the dose-dependent antibacterial ability of reduced GdW_10_O_36_ NCs against *E.coli* and *S.aureus* bacteria, a series of reduced GdW_10_O_36_ NCs at different concentrations was mixed with 500 μL of the as-prepared bacteria solution for 30 min. Finally, bacterial strains with a concentration of 10^3^ CFU∙mL^-1^ were cultured in solid medium for 24 h using the spread plate method to count the number of the bacterial colonies.

### *In vitro* antibacterial effect of reduced GdW_10_O_36_ NCs with peroxidase-like catalytic activity and photothermal therapy

To investigate the antibacterial property of peroxidase-like reduced GdW_10_O_36_ NCs with respect to *E.coli* and *S.aureus* bacteria, the following treatments were used on eight separate groups: (I) Control, (II) Reduced GdW_10_O_36,_ (III) H_2_O_2,_ (IV) Reduced GdW_10_O_36_ + H_2_O_2,_ (V) Control + NIR, (VI) Reduced GdW_10_O_36_ + NIR, (VII) H_2_O_2_ + NIR, and (VIII) Reduced GdW_10_O_36_ + H_2_O_2_ + NIR. The final concentration of bacteria was 1.0 × 10^6^ CFU mL^-1^, that of reduced GdW_10_O_36_ NCs was 200 μg mL^-1^, and that of H_2_O_2_ was 200 μM. After 10 min of co-incubation, groups V, VI, VII, and VIII were irradiated with an 808 nm NIR laser at a power density of 1.0 W cm^-2^ for 15 min. Then, an infrared thermal imager (E40, FLIR) was used to monitor the temperature. When co-incubation is conducted for 30 minutes, 100 μL of bacterial suspension in all of the groups was transferred to an agar culture plate for continuous cultivation for 12 h at 37 °C. All of the experiments were repeated in parallel three times.

### Fluorescent staining and SEM morphology observation of *E.coli* and *S.aureus* materials

Propidium iodide (PI) (Aladdin Company, China, Shanghai) was chosen to evaluate the dead fluorescent staining of bacteria. In the assay, *E.coli* and *S.aureus* bacteria were used in different experimental groups and were incubated with fluorescing propidium iodide (30 μM) at 25 °C (avoiding light) for 15 min. The stained bacterial suspension (5 μL) was dropped onto a microslide that was then covered a coverslip, and an inverted fluorescence microscope (Carl-Zeiss) was used to visualize the sample. For further visual observations of the specific morphology of the bacteria after different treatments, *E.coli* and *S.aureus* bacteria were obtained via centrifugation at 8,000 rpm for 1 min and fixed with 4.0 % glutaraldehyde solution overnight at 4 °C. After three repeated rinsings with PBS, a graded series of ethanol solutions (30, 50, 70, 80, 90, and 100 %) was used to dehydrate bacterial cells for 10 min. Finally, the obtained specimens were set on a silicon glide, sprayed with gold, and imaged under an ultrahigh-resolution field emission SEM (S-4700, Hitachi, Japan).

### Bacterial biofilm formation and antibacterial effects of reduced GdW_10_O_36_ NCs on bacterial biofilm

First, *E.coli* and *S.aureus* bacteria were washed three times with PBS (0.01 M) to discard the floating samples, and then 0.9 % saline solution was used to suspend the above-mentioned bacteria to an OD_600_ value up to 0.1. The diluted bacterial suspension (100 μL) was then co-incubated with 900 μL of LB or TSB medium for *E. coli* and *S. aureus* bacteria in a 96 well-plates. The samples were then continuously cultured and for 48 h at 37 °C. After that, fresh medium was substituted every 12 h. To evaluate the combined antibacterial effect of reduced GdW_10_O_36_ on bacterial biofilm, separate experiment groups were used: (I) PBS, (II) Reduced GdW_10_O_36_, (III) H_2_O_2_, (IV) Reduced GdW_10_O_36_ + H_2_O_2_, (V) PBS + NIR, (VI) Reduced GdW_10_O_36_ + NIR, (VII) H_2_O_2_ + NIR, and (VIII) Reduced GdW_10_O_36_ + H_2_O_2_ + NIR. After different disposes, bacterial biofilms were set aside in a shaker at 37 °C for 12 h. Then, 0.9 % physiological saline was used to rinse the biofilm three times to remove the samples and dead bacteria. Next, 500 μL of anhydrous alcohol was immediately used to fix the biofilm in each group, which was further cultured at 4 °C for 15 min. Thereafter, 300 μL of 1 % crystal violet staining solution was added to each well for 30 min of biofilm staining. Then, 0.01 M PBS was used to wash the biofilm in each well again, and the biofilms were then dried at room temperature. Last, each well was blended with anhydrous ethanol (500 μL) and co-incubated for 30 min. For the biofilm in each group, a multi-function microplate reader (Thermo Scientific, Varioskan LUK) was used to monitor the OD_590_ value, and a digital camera was used to record the color.

### Morphology analysis of biofilms using confocal laser scanning microscopy (CLSM)

For visually observations of biofilms using CLSM, biofilms were disposed with different methods. They were then cultured with Calcein-AM for 20 min and rinsed with 0.9 % NaCl to replace the discarded bacteriologic medium. An A1R-si Meta Inverted CLSM was then used to collect photographs with a size of 212.1 μm×212.1 μm and a framework size of 805×805. All of the samples were imaged with the same experimental facilities and analyzed using Zen 2011 software (Carl Zeiss). Each sample was independently measured in parallel three times.

### Animal study and wound healing process

First, female BALB/c mice (6-8 weeks, 18-23 g) were acquired from Beijing Vital River Laboratory Animal Technology Co., Ltd. and separated into six groups: (I) no treatment, (II) H_2_O_2_, (III) reduced GdW_10_O_36_ NCs, (IV) reduced GdW_10_O_36_ NCs + NIR, (V) H_2_O_2_ + reduced GdW_10_O_36_ NCs, and (VI) H_2_O_2_ + reduced GdW_10_O_36_ NCs + NIR with three mice in each group. Then, an *E.coli* (ATCC 6538) bacterial suspension (1×10^5^ CFU mL^-1^) was infected four times on each mouse's back to produce subcutaneous abscesses with a wound area of ~78 mm^2^ after anesthesia. Twelve hours later, 5 μL of reduced GdW_10_O_36_ NCs (100 μg/mL) and H_2_O_2_ solutions (100 μM) were added dropwise onto the subcutaneous abscess at each infected site in each corresponding group. The mouse in groups IV and VI was then further irradiated under an 808 nm NIR laser at a power density of 1.0 W cm^-2^ for 15 min; meanwhile, a thermal infrared imager was used to monitor temperature changes. Toward the end of all of the treatments, the wound site of each mouse was photographed every other day, and the wound tissues were collected and fastened with 4% paraformaldehyde solution for histological analysis with Hematoxylin and eosin (H&E) staining, Masson's trichrome staining and Giemsa staining.

## Results and Discussions

**Figure 1:**
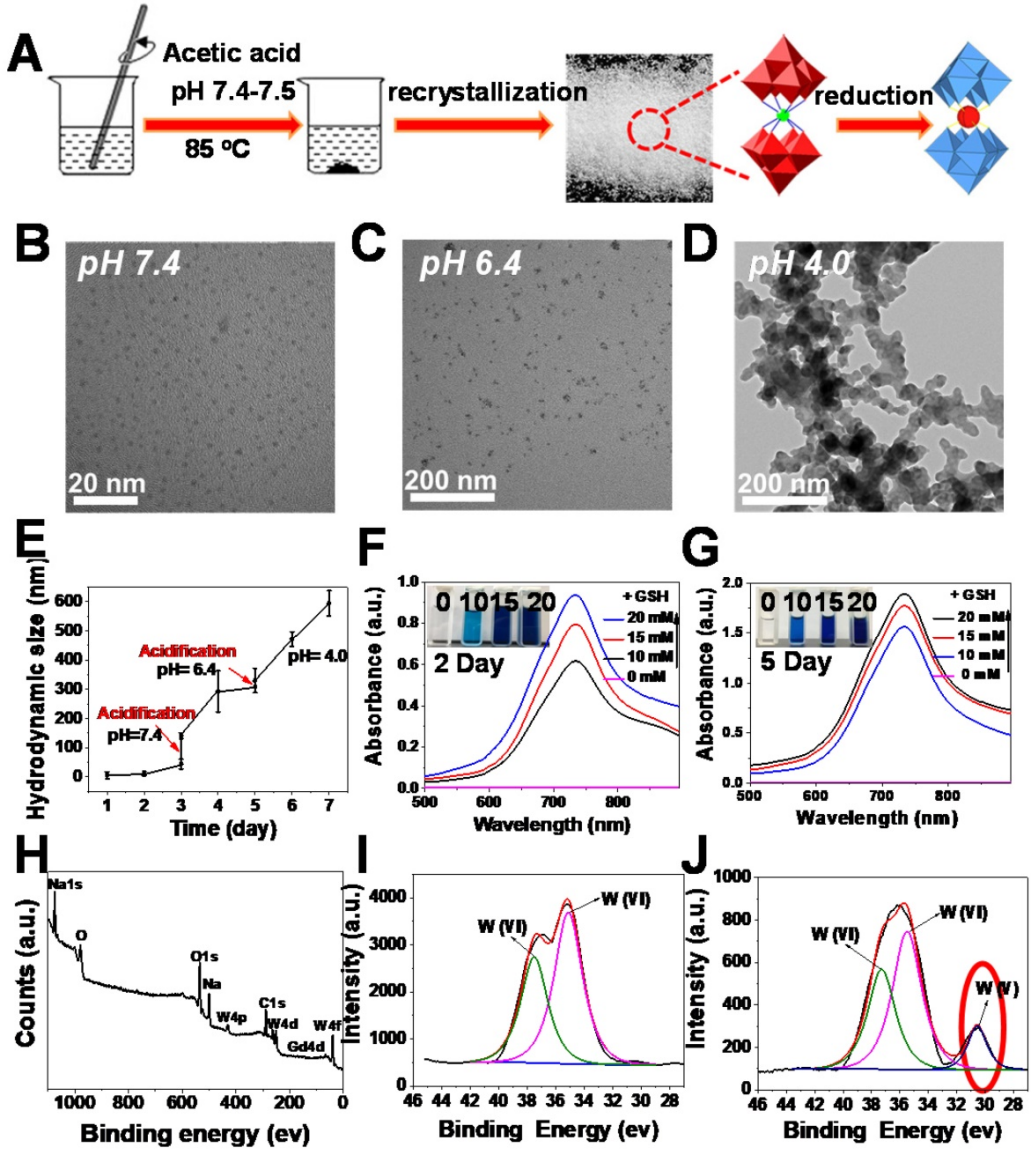
** Synthesis and characterization of oxidized and reduced GdW_10_O_36_ NCs.** (**A**) Schematic diagram of synthesis. (**B-D**) TEM images of nanoclusters at pH = 7.4, 6.4 and 4.0. (**E**) DLS measurements of GdW_10_O_36_ NCs with continuous acidification from pH = 7.4 to 4.0. UV-vis spectra and corresponding photographs of GdW_10_O_36_ clusters before and after reduction in the presence of various concentrations of GSH (0, 10, 15, and 20 mM) on the 2nd (**F**) and 5th day (**G**). (**H**) XPS spectra of GdW_10_O_36_ NCs. (**I-J**) XPS spectra of W 4f in GdW_10_O_36_ NCs before and after reduction.

To demonstrate the feasibility of our assumption, we first fabricated W-based polyoxometalate clusters (GdW_10_O_36_ NCs) according to a previously described method (Figure 1A) 59, 60. The GdW_10_O_36_ NCs were composed of cations (Na^+^ and NH_4_^+^) and Keggin-type macroanionic units. The successful fabrication was confirmed by Fourier transform infrared (FT-IR) spectroscopy and Raman spectroscopy (Figure S1). GdW_10_O_36_ NCs had a monodispersed spherical morphology and an ultrasmall diameter of about 1~3 nm that exhibited high hydrophilicity and dispersity at a pH of 7.4 (Figure 1B). Interestingly, as observed from transmission electron microscopy (TEM) (Figure 1C, D), these clusters exhibited distinct acidity-induced self-assembly with pH values that ranged from 7.4 to 4.0 when these clusters were acidified. They were homogeneously dispersed and had a diameter of ~ 30 nm in a slightly acidic environment (pH 6.4), and they further aggregated into a larger structured morphology when the pH was further decreased to 4.0. In particular, the acidity-activated self-assembly characteristic of GdW_10_O_36_ NCs was further confirmed by dynamic light scattering measurements (Figure 1E). The acidity-induced self-assembly of GdW_10_O_36_ NCs was ascribed to the protonation of POM macroanions through pH-induced hydrogen bond formation, which was beneficial for regulating the BME to achieve an enhanced antibacterial effect. Subsequently, to further investigate the redox-activated properties of the synthesized GdW_10_O_36_ NCs, glutathione (GSH) and ethylene glycol (EtOH), which are reducing agents, were used as patterns for incubating with GdW_10_O_36_ NCs at different concentrations. As seen in Figure 1F, G and S2, the pure solution of GdW_10_O_36_ NCs was colorless and had no absorption in the near infrared (NIR) region. However, the color of the solution gradually changed to blue after reduction with GSH and EtOH, This color change was simultaneously accompanied by a corresponding strong characteristic absorption in the NIR region, and this gradually became stronger with an increase in the GSH, EtOH, or GdW_10_O_36_ NCs concentration. Consistent with this, the relative residual amount of AA and GSH gradually decreased with GdW_10_O_36_ NCs oxidation, as shown in Figure S3, and this, further confirms the superior redox-activated properties of GdW_10_O_36_ NCs. Beyond that, to obtain fundamental insight into the redox-activated behavior of these POM clusters, X-ray photoelectron spectroscopy (XPS) was recorded. The XPS results reveal the oxidation state of W in GdW_10_O_36_ NCs before and after the reduction process. As seen in Figure 1H-J, GdW_10_O_36_ NCs were reduced by GSH to form reduced GdW_10_O_36_ NCs, and this confirms that the reduced GdW_10_O_36_ NCs were successfully synthesized. This phenomenon is the results of multiple reversible steps of electron exchange between delocalized electron density and occupiable cation site of W (V), which are present because of the increased reduction of GdW_10_O_36_ NCs 61, 62. This enhances the electron relaxation polarization and strengthens NIR absorption. Therefore, all of these results indicate that the GdW_10_O_36_ NCs have admirable acidic/reductive dual-responsive capability that enables the efficient regulation of the acidic BME and promotes oxidation of endogenous GSH, which play a critical role in the bacterial antioxidant defense system.

**Figure 2:**
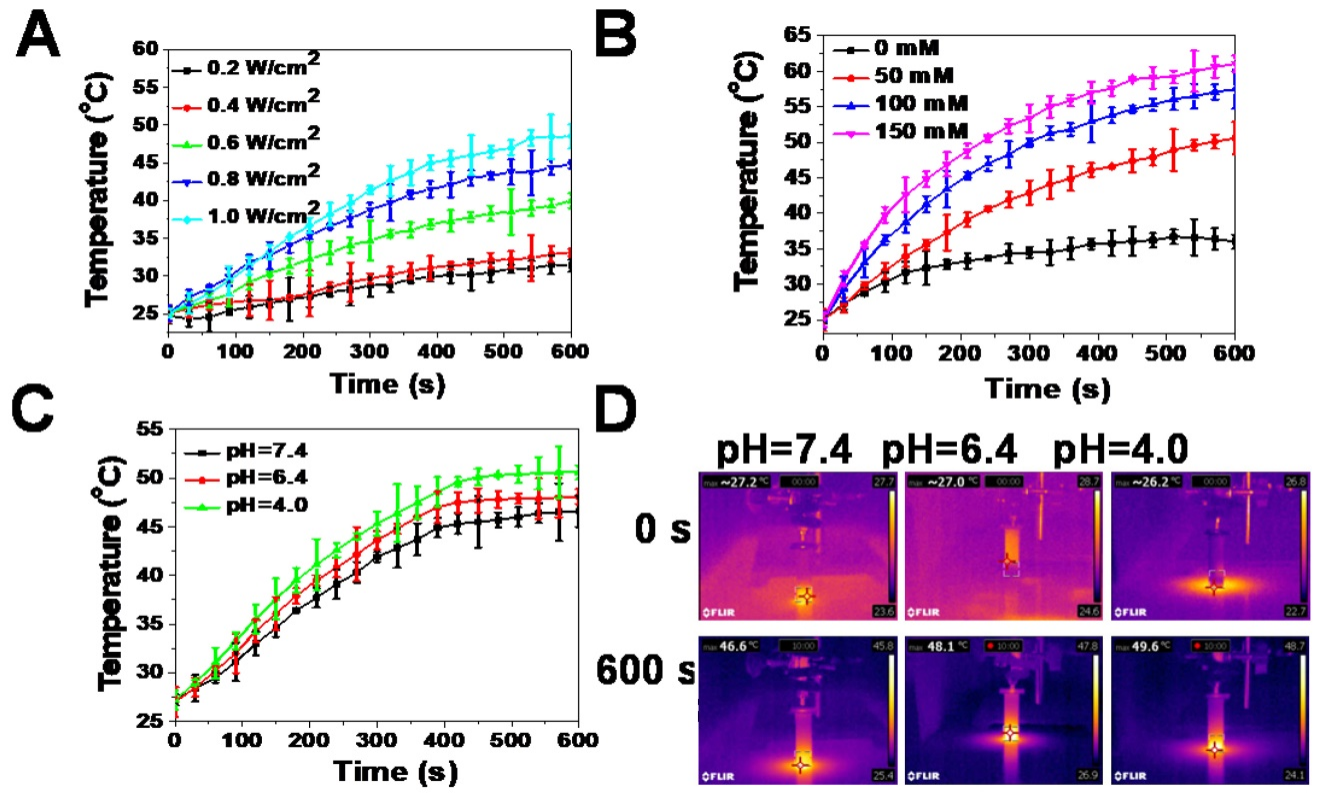
** Photothermal effects of reduced GdW_10_O_36_ NCs *in vitro*.** (**A**) Temperature evaluation of reduced GdW_10_O_36_ NCs (5.3 ppm Gd) as a function of irradiation time (0-10 min) using an 808 nm laser at different power densities (0.2, 0.4, 0.6, 0.8, and 1.0 W/cm^2^). (**B**) Temperature evaluation of reduced GdW_10_O_36_ NCs that were incubated with different concentrations of GSH (50, 100, and 150 mM) as a functional of irradiation time (0-10 min) using an 808 nm laser at a power density of 1.0 W/cm^2^. Acidity-dependent photothermal effects (**C**) and the corresponding IR images (**D**) of reduced GdW_10_O_36_ NCs (5.3 ppm Gd) under the irradiation of an 808 nm NIR laser at a power density of 1.0 W cm^-2^ for 10 min.

An aqueous solution of the reduced GdW_10_O_36_ NCs had a navy blue color and strong absorption in the NIR region. Thus, the photothermal effects of samples were carefully investigated. The results indicate that the photothermal effect of reduced GdW_10_O_36_ NCs depended on laser power intensity and GSH concentration. With an increase in the NIR laser power density, the temperature of this photothermal material increased (Figure 2A). Furthermore, after 10 min of irradiation under an NIR laser, GdW_10_O_36_ NCs that were mixed with different concentrations of GSH showed a higher increase in temperature. Moreover, GdW_10_O_36_ NCs that were co-incubated with 150 mM GSH at a pH of 7.4 even presented a remarkable temperature change of 36 ºC (Figure 2B). Interestingly, we found that the photothermal effect of reduced GdW_10_O_36_ NCs was also pH-dependent. This corresponds to low pH-induced self-assembly of GdW_10_O_36_ NCs and proportionally increases NIR absorbance (Figure 2C, D). The results showed that a more efficient increase in temperature can be monitored under BME-mimicked conditions under irradiation of an 808-nm laser, which facilitate to be a photothermal agent for photothermal antibacterial application. All of these results indicate that the obtained GdW_10_O_36_ NCs have the potential to be used for pH/GSH dual-responsive photothermal therapy.

**Figure 3:**
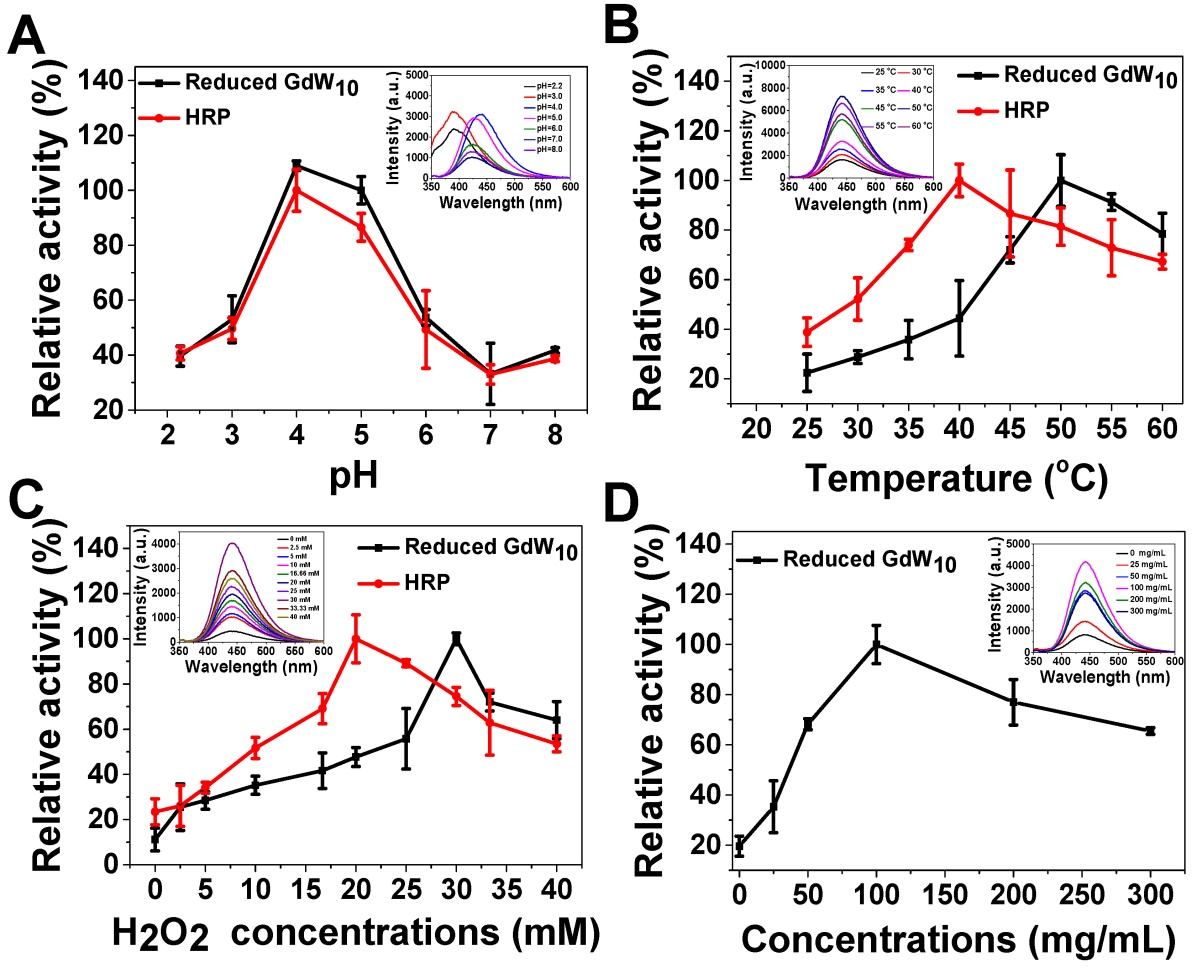
** Peroxidase-like activity of reduced GdW_10_O_36_ NCs is dependent on pH, temperature, H_2_O_2_, and concentrations**. (**A**) pH-dependent and (**B**) temperature-dependent activities with TA (0.5 mM), H_2_O_2_ (33 mM), and reduced GdW_10_O_36_ NCs (33 µg/mL). Reduced GdW_10_O_36_ NCs and HRP show an optimal pH of 4.0-5.0 and optimal temperature around 40-50 °C. (**C**) H_2_O_2_ concentration-dependent peroxidase-like activity with reduced GdW_10_O_36_ NCs (33 µg/mL) and TA (0.5 mM). Reduced GdW_10_O_36_ NCs require a higher H_2_O_2_ concentration than HRP to reach maximal peroxidase activity. (**D**) Reduced GdW_10_O_36_ NCs-dependent peroxidase-like activity with TA (0.5 mM) and H_2_O_2_ (33 mM). The maximum point in each curve (A-D) was set as 100 %. The insets show the fluorescence spectra of the corresponding reduced GdW_10_O_36_ NCs reaction system.

Furthermore, one of the unique features of our GdW_10_O_36_ NCs system is that, except for intelligent acidic/reductive dual-responsive enhanced photothermal efficacy under BME-mimicked conditions; the GdW_10_O_36_ NCs after reduction could act simultaneously as a robust and effective intrinsic peroxidase mimic for catalyzing bacterial endogenous H_2_O_2_ to achieve enhanced biofilm elimination and favorable antibacterial effects. In the following, we employed terephthalic acid (TA) and 3,3,5,5-tetramethylbenzidine (TMB) as peroxidase substrates for evaluating the peroxidase-like activity of reduced GdW_10_O_36_ NCs or the commonly used enzyme HRP. The results indicate that reduced GdW_10_O_36_ NCs and HRP can both efficiently catalyze •OH radical generation to form a strong fluorescence intensity with TA or a characteristic absorption peak at 652 nm with the TMB substrate (Figure 3); with 33 mM of H_2_O_2_, The system was blue (Figure S4). Simultaneously, like other peroxidase mimics, the catalytic activity of reduced GdW_10_O_36_ NCs, was dependent on pH, temperature, H_2_O_2_ and concentrations; these characteristics are similar to those of HRP. Notably, the high activity of reduced GdW_10_O_36_ NCs and HRP was optimal at a pH of 5.5, which is approximately the acidic BEM (Figure 3A). Hence, the enzyme mimics that have high catalytic activity in the important pH range of 3.5-5.5 (i.e., the pH of an acidic BME) will be promising candidates for use in biological and antibacterial research. Additionally, we discovered that compared to HRP, reduced GdW_10_O_36_ NCs can withstand higher temperature and higher H_2_O_2_ concentration to achieve maximum values of peroxidase activity. Nevertheless, the peroxidase-like activity was suppressed if the temperature were further elevated or if the H_2_O_2_ concentration were further increased, as indicated by the enzymic catalytic reaction (Figure 3B-D). Thus, all of the above results demonstrate that reduced GdW_10_O_36_ NCs can serve as peroxidase mimics that have high activities and stability for enhancing biofilm removal and for facilitating antibacterial effects.

**Figure 4:**
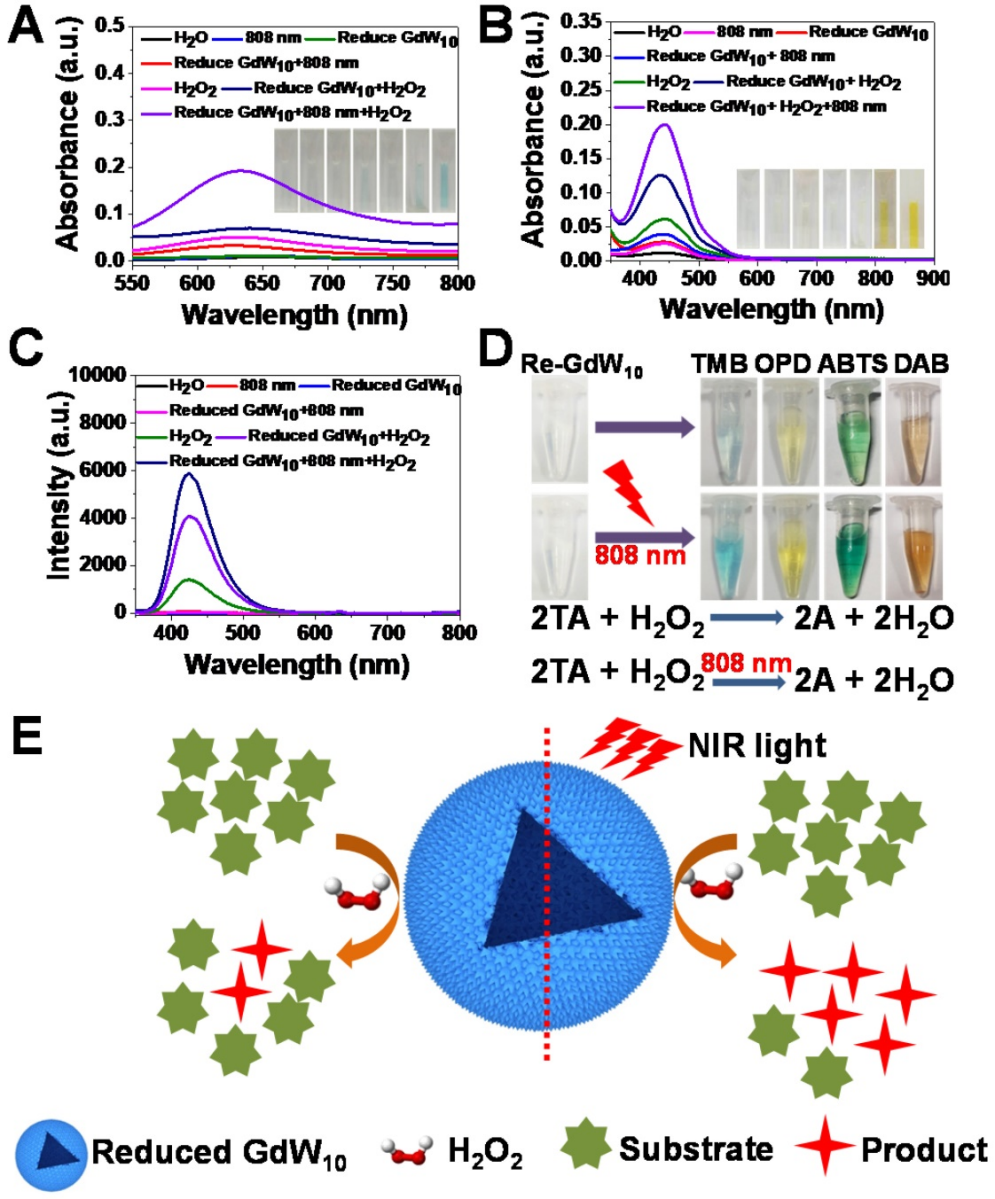
** Enzyme-like properties of reduced GdW_10_O_36_ NCs.** (**A, B**) Absorbance spectra and visual color changes of TMB (1 mM) and OPD (1.85 mM) in different reaction systems: (1) H_2_O+TMB, (2) 808 nm+TMB, (3) Reduced GdW_10_O_36_ NCs+TMB, (4) Reduced GdW_10_O_36_ NCs+808 nm+TMB, (5) H_2_O_2_+TMB, (6) Reduced GdW_10_O_36_ NCs+H_2_O_2_+TMB, and (7) Reduced GdW_10_O_36_ NCs+808 nm+H_2_O_2_+TMB. (**C**) Fluorescence spectra of terephthalic acid for hydroxyl radical detection at the maximum emission wavelength of 435 nm. (**D**) Different color reactions and the mechanism scheme of reduced GdW_10_O_36_ NCs catalysis in the presence of H_2_O_2_ and various peroxidase substrates represented by AH. (**E**) Scheme of the catalytic mechanism of reduced GdW_10_O_36_ NCs combined with irradiation from an 808 nm laser in the presence of H_2_O_2_.

To further understand the fundamental catalytic mechanism of reduced GdW_10_O_36_ NCs, we tested whether the reduced GdW_10_O_36_ NCs can promote the decomposition of H_2_O_2_ into hydroxyl radicals (•OH) under the irradiation of an 808 nm laser to improve antibacterial activities for synergitic wound therapy. Thus, we used 3,3,5,5-tetramethylbenzidine (TMB), OPD and TA as substracts to record the production of hydroxyl radicals (•OH) that originate from the effective photothermal conversion of the reduced GdW_10_O_36_ NCs to promote the low concentration of H_2_O_2_ species decomposition. As expected, the fluorescence intensity is significantly enhanced in the presence of reduced GdW_10_O_36_ NCs compared the fluorescence intensity of the pure H_2_O_2_ species. This indicates that reduced GdW_10_O_36_ NCs powerful catalysts for the prodution of •OH from H_2_O_2_ because of their optimal peroxidase-like catalytic activity. More interestingly, when reduced GdW_10_O_36_ NCs were treated successively with H_2_O_2_ for 10 min and irradiation from an 808 nm laser for 30 min, an apparent increase in fluorescence intensity was monitored in this system. In contrast, only a relative weak fluorescence signal was obtained in the absence of either H_2_O_2_ or irradiation from an 808 nm laser (Figure 4A-C). This phenomenon was also shown in the enzyme-catalyzed reactions (Figure 4D). A clearer explanation of the aforementioned results is described below for the brief enhancement mechanism of reduced GdW_10_O_36_ NCs that are irradiated with an 808 nm laser to produce •OH radicals (Figure 4E). Therefore, these results effectively confirm that reduced GdW_10_O_36_ NCs significantly enhance the formation of •OH species via the combination of their photothermal effect and peroxidase-like catalytic activity.

**Figure 5:**
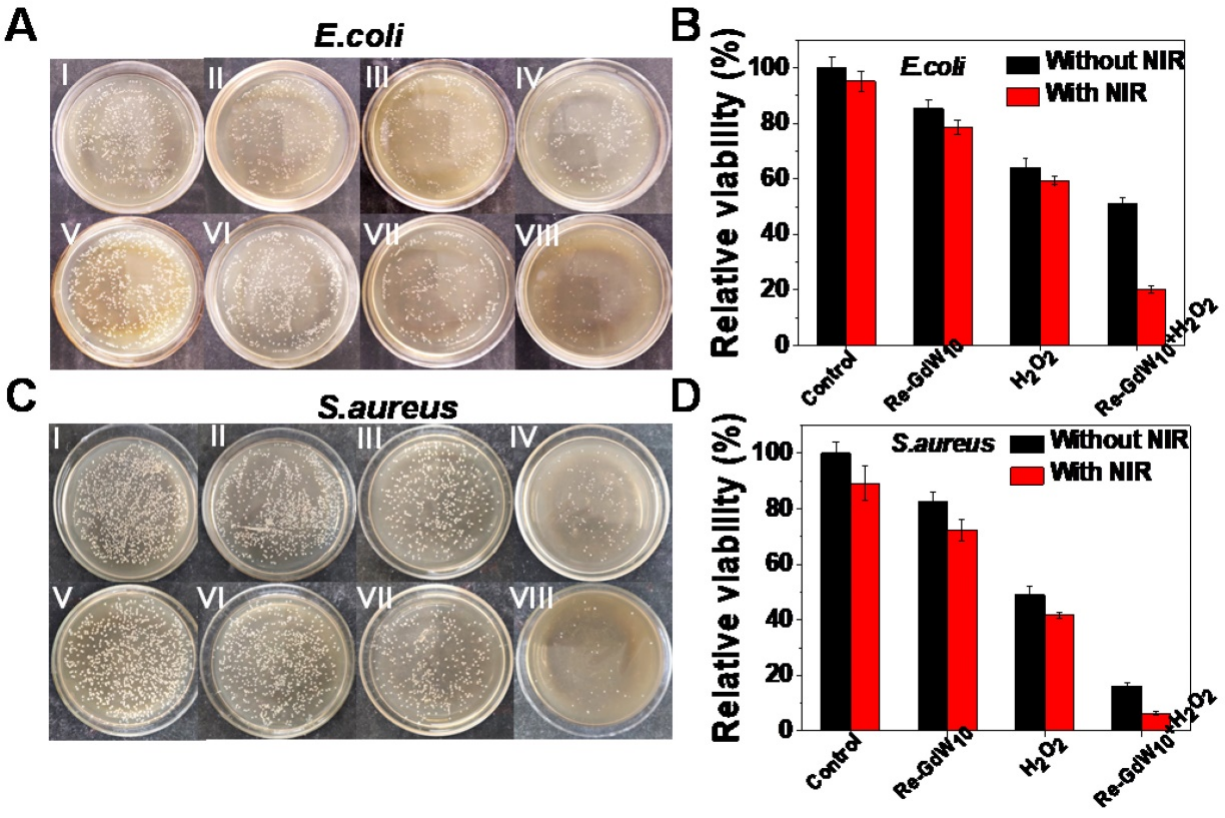
** Photographs and antibacterial activity of reduced GdW_10_O_36_ NCs after different treatments.** (A, C) Photographs and (B, D) survival rates of treated *E. coli* and *S.aureus* after exposed to (I) Control, (II) Reduced GdW_10_O_36_, (III) H_2_O_2_, (IV) Reduced GdW_10_O_36_+ H_2_O_2_, (V) Control+NIR, (VI) Reduced GdW_10_O_36_+NIR, (VII) H_2_O_2_ +NIR, and (VIII) GdW_10_O_36_ + H_2_O_2_ +NIR. Conditions: 200 µg/mL reduced GdW_10_O_36_, 200 µM H_2_O_2_, 1 W/cm^2^ 808 nm laser and 15 min. P values were based on the Student's test: *P < 0.05, and **P < 0.01.

To demonstrate the antibacterial effect of the reduced GdW_10_O_36_ NCs-based biocatalytic process under pathological conditions, a peroxidase-mediated antibacterial assessment against *E. coli* or and *S. aureus* bacteria was carried out using the standard colony counting method to explore the feasibility of reduced GdW_10_O_36_ NCs as peroxidase mimics and photothermal agents to enhance the generation of reactive oxygen species. After incubation with large concentrations of reduced GdW_10_O_36_ NCs for 24 h, no obvious reduced bacteria population and cells were observed in *E. coli* and *S. aureus* bacteria as well as 4T1 cells, even at a dose as high as 1 mg/mL (Figures S5 and S6). This indicates that reduced GdW_10_O_36_ NCs with superior biocompatibility and low cytotoxicity can meet the requirements for biomedical applications. Next, we explored the antibacterial effects of these NCs as simultaneous peroxidase and photothermal agents. As seen in Figure 5A, B, significantly reduced numbers of colonies were observed after bacteria were incubated with reduced GdW_10_O_36_ NCs in the presence of H_2_O_2_ for 10 min and then irradiated with an 808 nm NIR laser for 15 min. Specifically, the bacteria proliferation inhibition percentages reached 80% for* E. coli* bacteria. In contrast, in the absence of either H_2_O_2_ or irradiation under an 808 nm laser, the reduced GdW_10_O_36_ NCs group had relatively inferior antibacterial behavior. Meanwhile, because of the low concentration of H_2_O_2_ (200 μM) and reduced GdW_10_O_36_ concentration (200 μg/mL), the single H_2_O_2_ group and reduced GdW_10_O_36_ NCs group both exhibited negligible antibacterial effects towards *E. coli.* The survival percentages were 85.4% and 64.1%, respectively, which indicates that peroxidase catalysis alone cannot achieve ideal antibacterial effects. Correspondingly, similar experimental results were also confirmed in *S. aureus* bacteria (Figure 5C, D). Consequently, all of the results further substantiate that reduced GdW_10_O_36_ NCs have a superior capacity to be function as peroxidase-like enzymes and photothermal agents with an effective combined antibacterial application.

**Figure 6:**
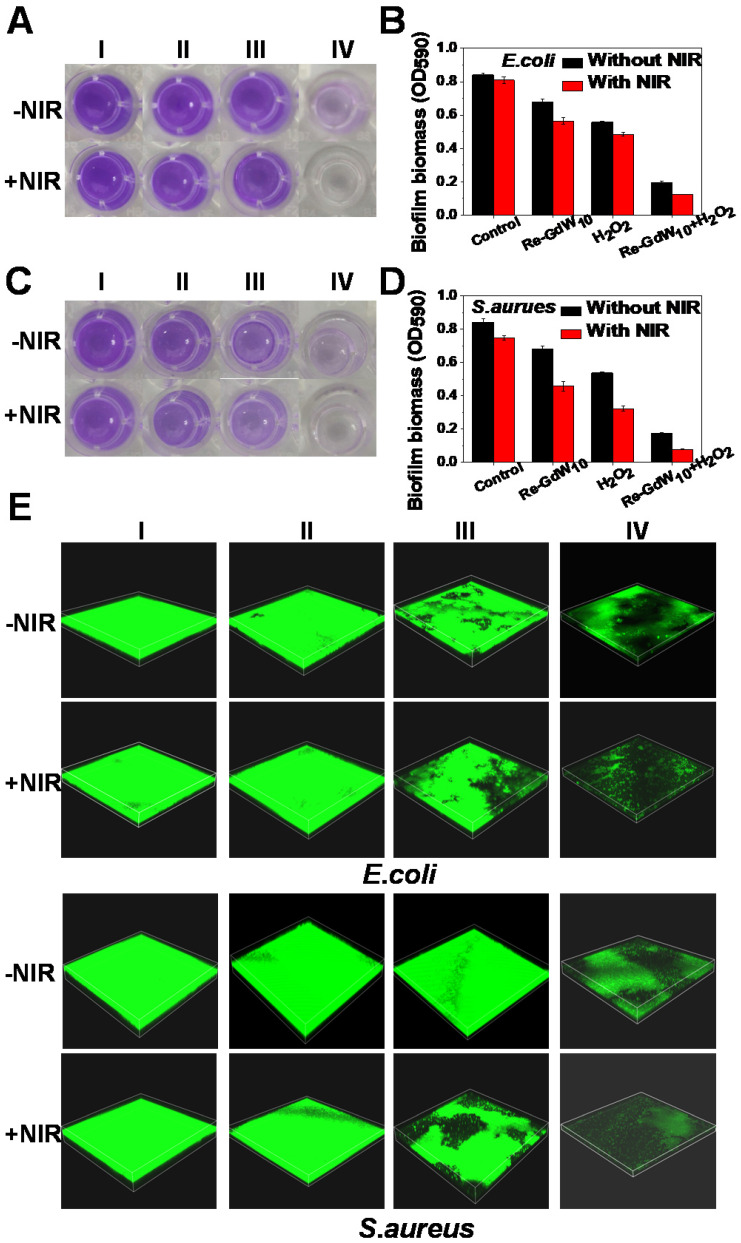
** Bacterial biofilm elimination effect and images of reduced GdW_10_O_36_ NCs after different treatments.** (A, C) Bacterial biofilm eradication effect of *E. coli* and *S.aureus* bacteria treated with reduced GdW_10_O_36_ NCs after after treated with (I) PBS (control), (II) Reduced GdW10, (III) H_2_O_2_, (IV) Reduced GdW_10_ + H_2_O_2_ in the presence or absence of NIR. (B, D) OD_590_ value for evaluating changes in biofilm biomass of *E. coli* and *S.aureus* bacteria after different treatments. (E) 3D confocal laser scanning microscopy (CLSM) of biofilms formed from *E. coli* and *S.aureus* bacteria treated with reduced GdW_10_O_36_ NCs after different treatments. Image sizes: 212.1×212.1 µm. The biofilms were stained with CA. P values were based on the Student's test: *P < 0.05, and **P < 0.01.

**Figure 7:**
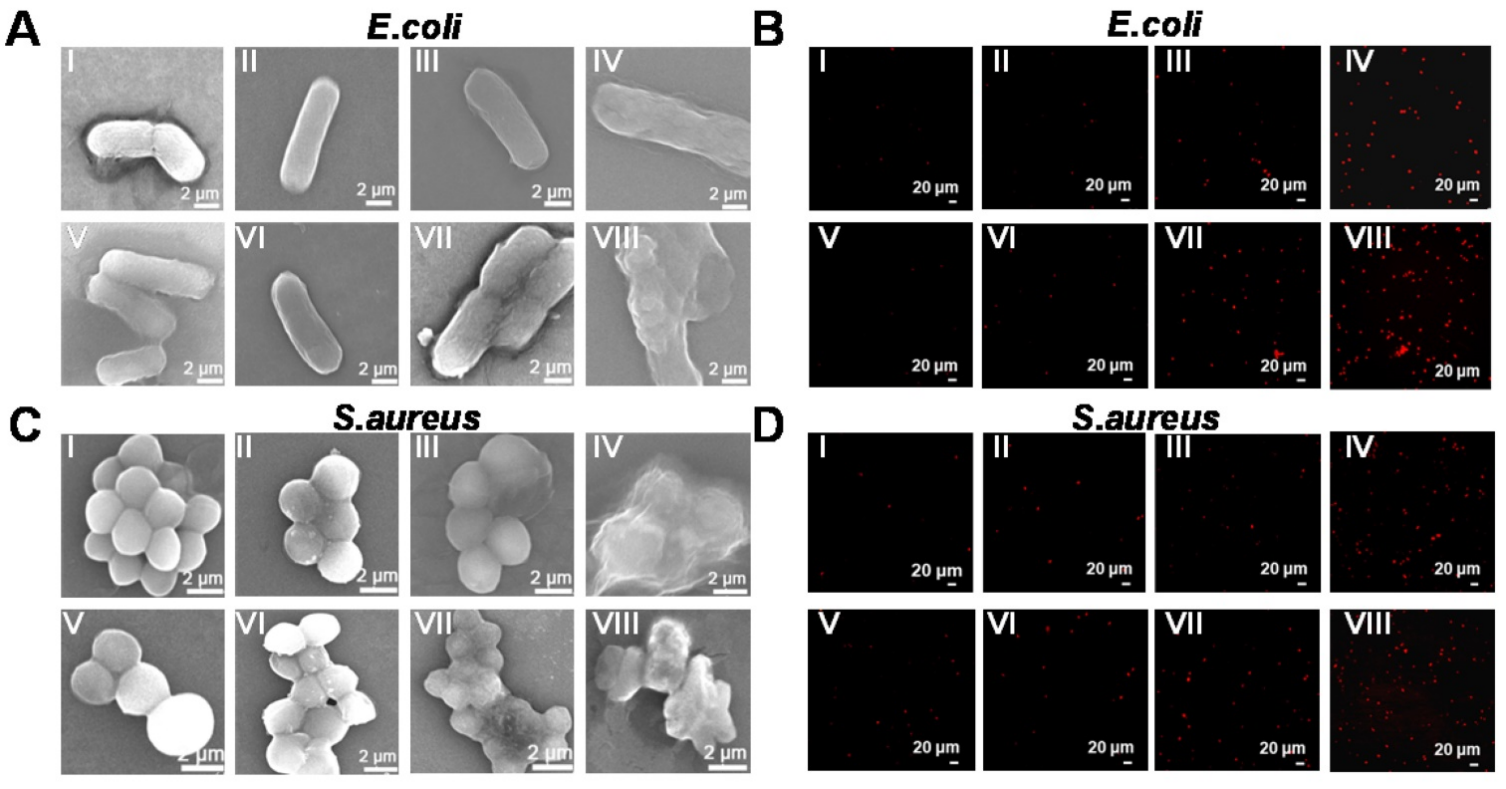
** Morphology and live/dead staining imaging of *E. coli* and *S. aureus* bacteria after different treatments.** SEM images (A, C) and fluorescent staining photographs (B, D) of *E. coli* and *S. aureus* display bacterial membrane changes after different treatments. (I) Control, (II) Reduced GdW_10_O_36_, (III) H_2_O_2_, (IV) Reduced GdW_10_O_36_+H_2_O_2_, (V) Control+NIR, (VI) Reduced GdW_10_O_36_+NIR, (VII) H_2_O_2_ +NIR, and (VIII) GdW_10_O_36_ + H_2_O_2_ +NIR. (A, C: Scale bar represents 2 µm; B, D: Scale bar represents 20 µm).

Additionally, the biofilm, which is a hybrid of bacterial cells, irreversibly adheres onto the surface of a material or tissue. It is packed in self-generated extracellular polymeric substances (EPS), which act as a barrier in preventing antibiotic permeation into the bacterial cells; consequently, bacterial drug-resistance is produced and antibiotic treatment failure occurs. Therefore, anti-biofilm activity is an important characteristic of antibacterial agents for achieving desirable antibacterial effects. Herein, the crystal violet staining method was used to investigate biofilm formation of reduced GdW_10_O_36_ NCs after different treatments. As seen in Figure 6A, C, the navy blue color remained in the reduced GdW_10_O_36_ NCs, H_2_O_2_ and 808-nm laser alone treated groups. A slight decrease in the blue color was observed for the groups that were treated with the combination of reduced GdW_10_O_36_ NCs plus laser or the combination of reduced GdW_10_O_36_ NCs and H_2_O_2_. Nevertheless, the blue color significantly faded to colorless after the combined treatment of reduced GdW_10_O_36_ NCs with laser irradiation or with H_2_O_2_ treatment. Accordingly, biofilm biomass of *E. coli* bacteria that were treated with different methods was also quantified using UV-vis spectroscopy (Figure 6B, D). The results indicate that much less biofilm biomass are measured for disposed with reduced GdW_10_O_36_ NCs and laser irradiation in the presence of H_2_O_2_ in comparison to the monotherapy groups or the control group. Furthermore, 3D confocal microscopy was used to analyze the average thickness of biofilms that formed on glasses via different treatments. As seen in Figure 6E&S7, much thinner biofilms formed on reduced GdW_10_O_36_ NCs and H_2_O_2_ as well as for the samples that were irradiated with a laser. In contrast, much thicker biofilms were observed on the surfaces of samples in the monotherapy groups or control group. These observations confirm that peroxidase-like catalysis and the photothermal effect of reduced GdW_10_O_36_ NCs more efficiently reduce the thickness of biofilm. This phenomenon is ascribed to the unique electrical structure and acidity of reduced GdW_10_O_36_ NCs for regulating the BME and achieving antibacterial effects. We also used scanning electron microscopy to observe material adsorption on bacterial membranes and to investigate changes in the morphology of treated *E. coli* and *S. aureus*. As seen in Figure 7A, C, the negative group exhibited smooth, rod-like and sphere-like shapes, and the surface was intact. In contrast, membranes of bacteria distorted and shrunk to some extent after the sample was irradiated with an 808 nm laser or exposed to a low concentration of H_2_O_2_ alone. Moreover, we found that bacteria membranes were seriously destroyed and crashed after the combined photothermal and H_2_O_2_ treatment, and this was attributed to the strong production of •OH that was induced by reduced GdW_10_O_36_ NCs and H_2_O_2_ under 808 nm laser exposure. In addition, we used live/dead staining imaging to demonstrate the relative survival of bacteria after different treatments (Figure 7B, D). The results were similar to the previously mentioned bacterial cloning and scanning electron microscopy results, and this further confirms that reduced GdW_10_O_36_ NCs have an admirable capacity for efficiently inhibiting bacterial proliferation because of their photothermal and peroxidase-like catalysis.

**Figure 8:**
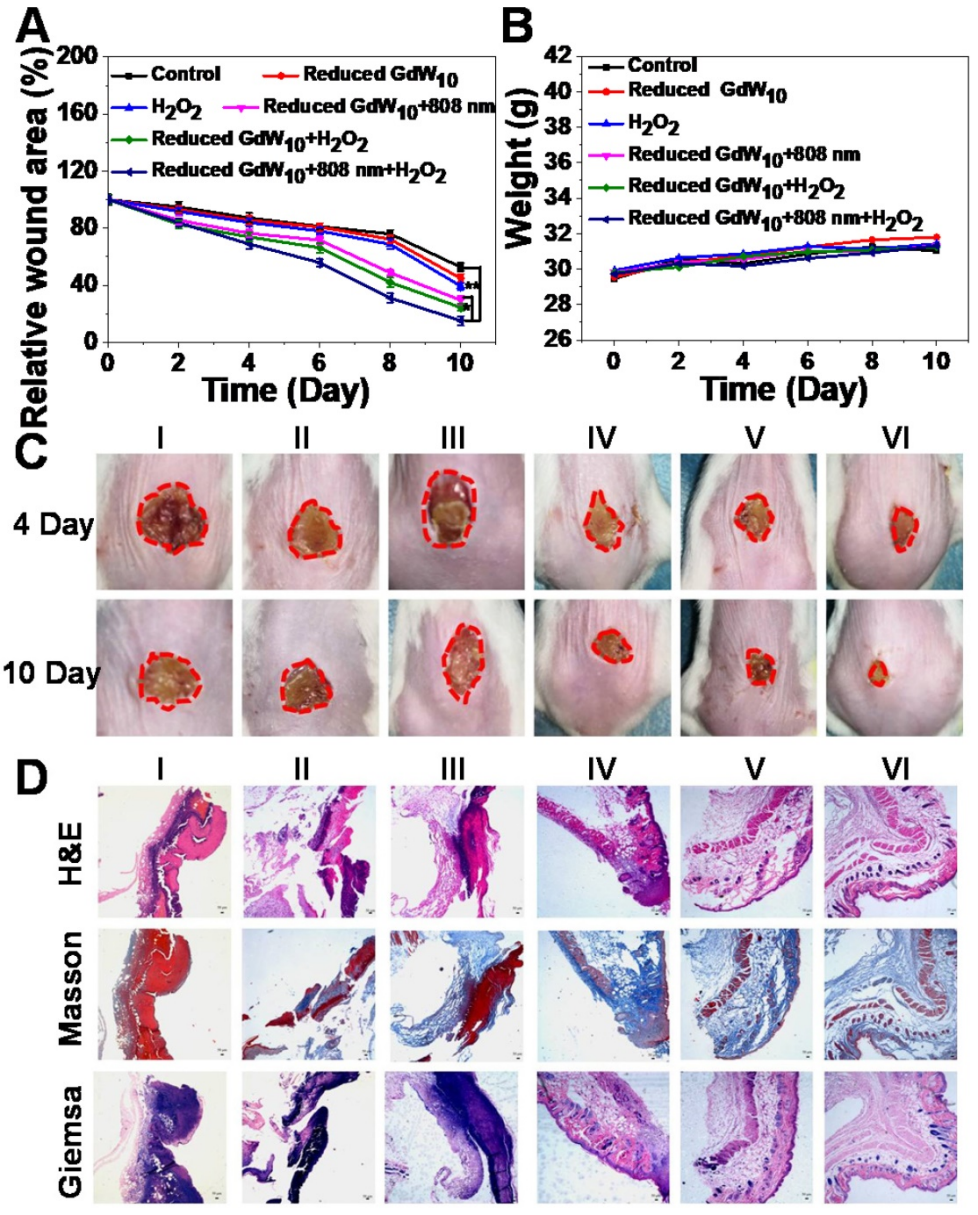
** Wound-healing studies of reduced GdW_10_O_36_ NCs after different treatments *in vivo***. (A) Relative wound area and (B) weight of mice infected with *E.coil* after different treatments. (C) Photographs and histologic analyses (D) of *E. coli-* infected wound treated with (I) PBS (control), (II) H_2_O_2_ , (III) Reduced GdW_10_, (IV) Reduced GdW_10_ +NIR, (V) Reduced GdW_10_ +H_2_O_2_, and (VI) Reduced GdW_10_ +NIR+H_2_O_2_ at days 4 and 10 and their corresponding histologic analyses. P values were based on the Student's test: *P < 0.05, and **P < 0.01.

To further investigate the potential that reduced GdW_10_O_36_ NCs with antibacterial activity have for promoting wound healing *in vivo*, *E. coli* bacteria-infected wound-disinfected mice were first designed and then divided into six groups:(I) PBS, (II) H_2_O_2_, (III) Reduced GdW_10_, (IV) Reduced GdW_10_ + NIR, (V) Reduced GdW_10_ + H_2_O_2_, and (VI) Reduced GdW_10_ + NIR + H_2_O_2_. The concentration of reduced GdW_10_O_36_ NCs was fixed at 100 μg/mL, and that of H_2_O_2_ was fixed at 100 μM; these are much less than the concentrations that are commonly used (0.5-3 % and about 166 mM to 1 M) 38-40. During therapeutic periods, the states of wounds were photographed on days of 4 and 10. As seen in Figure 8A, B, the wounds in the group without treatment and in the group that received reduced GdW_10_O_36_ NCs or H_2_O_2_ alone exhibited serious inflammatory reactions with ulceration, erythema, and edema on day 4. Also, the relative wound area was only reduced 81.54±3.09%, 84.48±3.69% and 75.94±3.63%, respectively. In contrast, the group that treated with reduced GdW_10_O_36_ NCs in the presence of irradiation with an 808 nm laser or with H_2_O_2_ alone, the relative wound area only decreased to 71.88±2.81% and 70.79±3.77%, respectively. This indicates that there was a weak inhibition effect of photothermal or peroxidase-like catalytic effect alone that was induced by reduced GdW_10_O_36_ NCs. Surprisingly, the reduced GdW_10_O_36_ NCs + NIR + H_2_O_2_-treated group was effective against wound infection and effectively accelerated wound healing and scab formation after 4 days of treatment. Also, the wound was sharply crusted, and the relative area was reduced to 9.18±2.70% after 10 days of treatment. Accordingly, histological analysis containing hematoxylin and eosin and masson and giemsa staining revealed that the level of inflammation in the abscesses that were treated with reduced GdW_10_O_36_ NCs in the presence of irradiation from an 808 nm laser or of H_2_O_2_ alone slightly decreased over time, which is contrary to the untreated group. More importantly, the skin of the mice was almost completely recovered on day 10 after treatment with reduced GdW_10_O_36_ NCs + NIR + H_2_O_2_ (Figure 8C). Meanwhile, regeneration and differentiation of fibroblasts under the dermis as well as regeneration of the skin around the wound are important processes in wound healing. Therefore, structure changes to the epidermis and formation of collagen fiber during wound healing were further investigated. Hematoxylin and eosin (H&E) and Masson and Giemsa staining of the epidermis of repaired tissues after different treatments were performed on day 10. As seen in Figure 8D, H&E staining indicates that new fibroblasts and newborn blood vessels filled with plenty of red blood cells had begun to move to the wound position from adjacent normal tissue or organ for the reduced GdW_10_O_36_ + H_2_O_2_ + NIR group by the end of the experiment. In contrast, abundant dead cell fragments and a loose disordered incomplete dermal layer are still observed in other groups. Furthermore, Masson's trichrome staining showed infected necrotic foci, edema, ulceration, and some inflammatory cells were also seen in the control groups. Also, the skin epidermis structure and collagen fiber of the monotherapy group (reduced GdW_10_O_36_ + H_2_O_2_ and reduced GdW_10_O_36_ + NIR) are relatively clear, complete, and regenerated. In contract, intact established collagen fibers and dermal layer were observed in the reduced GdW_10_O_36_ + H_2_O_2_ + NIR group, and this implies that the wounds have completely healed. Additionally, Giemsa staining was used to test the amounts of adherent bacteria after 10 days of treatments. The results indicate that the amount of bacteria in the reduced GdW_10_O_36_ + H_2_O_2_ + NIR group decreased significantly in comparison to the control and monotherapy groups. This confirms the positive effects of reduced GdW_10_O_36_ during wound treatment. In brief, this antibacterial platform efficiently inhibits bacterial proliferation, and also promotes differentiation of fibroblasts and regeneration of skin. Therefore, all of these results demonstrated that reduced GdW_10_O_36_ NCs have great potential for accelerating wound healing in the *E. coli*-infected abscesses because of their superior photothermal efficiency and surprisingly high peroxidase-like activity.

## Conclusions

In brief, we successfully developed an antimicrobial platform using the intelligent tungsten (W)-polyoxometalate cluster paradigm. This system features pH/GSH dual-response via acidic BME-triggered self-assembly and reductive BME-driven strengthened NIR absorption. Thus, it results in the rapid elimination of biofilm and exhibits remarkable antibacterial activity upon exposure to NIR laser radiation. Additionally, the intrinsic peroxidase-mimic activity of reduced POMs that are generate in a BME can catalyze the decomposition of endogenous H_2_O_2_ with low concentrations to generate·OH. More importantly, in the presence of bacterial endogenous H_2_O_2_, an enhanced antibacterial effect can be achieved via the strengthened photothermal effect and intrinsic peroxidase-mimic activity of reduced POMs. Therefore, the intelligent pH/GSH dual-responsive tungsten (W)-polyoxometalate cluster resolves the problem of severe biofilm infections and also achieves an unprecedented antibacterial efficacy. In brief, the particular acidic/reductive dual-responsiveness of POMs is anticipated to be a powerful tool for illustrating significant guidance on the future design of BME-specific antibacterial nanoplatforms with efficient antibacterial effects.

## Supplementary Material

Supplementary figures.

## Figures and Tables

**Scheme 1:**
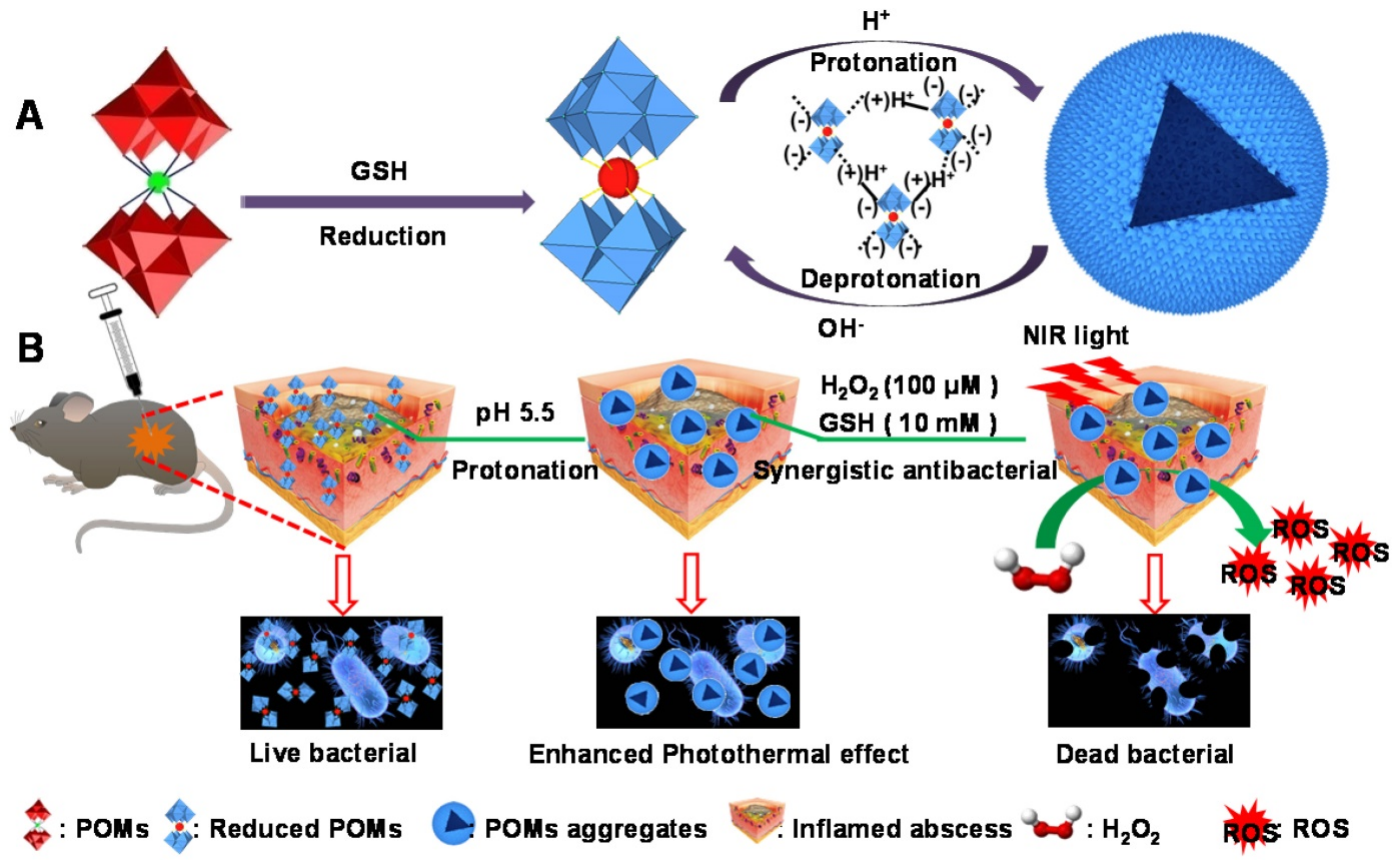
Schematic illustration of pH/GSH responsiveness of GdW_10_O_36_ NCs as a combined paradigm for peroxidase catalyst-photothermal effect to regulate BMEs for enhancing antibiofilm activity and wound healing.

## References

[1] Fair RJ, Tor Y (2014). Antibiotics and bacterial resistance in the 21st century. Perspect Medicin Chem.

[2] Fang G, Li W, Shen X, Perez-Aguilar JM, Chong Y, Gao X (2018). Differential Pd-nanocrystal facets demonstrate distinct antibacterial activity against gram-positive and gram-negative bacteria. Nat Commun.

[3] Koo H, Allan RN, Howlin RP, Stoodley P, Hall-Stoodley L (2017). Targeting microbial biofilms: current and prospective therapeutic strategies. Nat Rev Microbiol.

[4] Li P, Poon YF, Li W, Zhu HY, Yeap SH, Cao Y (2011). A polycationic antimicrobial and biocompatible hydrogel with microbe membrane suctioning ability. Nat Mater.

[5] Teng CP, Zhou T, Ye E, Liu S, Koh LD, Low M (2016). Effective targeted photothermal ablation of multidrug resistant bacteria and their biofilms with NIR-absorbing gold nanocrosses. Adv Healthc Mater.

[6] Abee T, Kovacs AT, Kuipers OP, Van Der Veen S (2011). Biofilm formation and dispersal in gram-positive bacteria. Curr Opin Biotechnol.

[7] Hu D, Li H, Wang B, Ye Z, Lei W, Jia F (2017). Surface-adaptive gold nanoparticles with effective adherence and enhanced photothermal ablation of methicillin-resistant staphylococcus aureus biofilm. ACS Nano.

[8] Costerton JW, Stewart PS, Greenberg EP (1999). Bacterial biofilms: a common cause of persistent infections. Science.

[9] Fabrega J, Renshaw JC, Lead JR (2009). Interactionsof silver nanoparticles with pseudomonas putida biofilms. Environ Sci Technol.

[10] Durmus NG, Taylor EN, Kummer KM, Webster TJ (2013). Enhanced efficacy of superparamagnetic iron oxide nanoparticles against antibiotic-resistant biofilms in the presence of metabolites. Adv Mater.

[11] Wang X, Wu J, Li P, Wang L, Zhou J, Zhang G (2018). Microenvironment-responsive magnetic nanocomposites based on silver nanoparticles/gentamicin for enhanced biofilm disruption by magnetic field. ACS Appl Mater Interfaces.

[12] Wang CL, Chen P, Qiao YB, Kang Y, Yan CR, Yu Z (2020). pH responsive superporogen combined with PDT based on poly Ce6 ionic liquid grafted on SiO_2_ for combating MRSA biofilm infection. Theranostics.

[13] Bae K, Zheng W, Ma Y, Huang ZW (2019). Real-time monitoring of pharmacokinetics of antibiotics in biofilms with Raman-tagged hyperspectral stimulated Raman scattering microscopy. Theranostics.

[14] Davies DG, Marques CN (2009). A fatty acid messenger is responsible for inducing dispersion in microbial biofilms. J Bacteriol.

[15] Lu MM, Ge Y, Qiu J, Shao D, Zhang Y, Bai J (2018). Redox/pH dual-controlled release of chlorhexidine and silver ions from biodegradable mesoporous silica nanoparticles against oral biofilms. Int J Nanomedicine.

[16] Li X, Wu B, Chen H, Nan K, Jin Y, Sun L (2018). Recent developments in smart antibacterial surfaces to inhibit biofilm formation and bacterial infections. J Mater Chem B.

[17] Ninan N, Forget A, Shastri VP, Voelcker NH, Blencowe A (2016). Antibacterial and anti-inflammatory pH-responsive tannic acid-carboxylated agarose composite hydrogels for wound healing. ACS Appl Mater Interfaces.

[18] Xiong MH, Li YJ, Bao Y, Yang XZ, Hu B, Wang J (2012). Bacteria-responsive multifunctional nanogel for targeted antibiotic delivery. Adv Mater.

[19] Finnegan M, Linley E, Denyer SP, McDonnell G, Simons C, Maillard JY (2010). Mode of action of hydrogen peroxide and other oxidizing agents: differences between liquid and gas forms. J Antimicrob Chemother.

[20] Yin W, Yu J, Lv F, Yan L, Zheng LR, Gu Z (2016). Functionalized nano-MoS_2_ with peroxidase catalytic and near-infrared photothermal activities for safe and synergetic wound antibacterial applications. ACS Nano.

[21] Wang B, Feng GX, Seifrid M, Wang M, Liu B, Bazan GC (2017). Antibacterial narrow band gap conjugated oligoelectrolytes with high photothermal conversion efficiency. Angew Chem Int Ed Engl.

[22] Xu BL, Wang H, Wang WW, Gao LZ, Li SS, Pan XT (2019). Single-atom nanozyme for wound antibacterial applications. Angew Chem Int Ed Engl.

[23] Dai X, Zhao Y, Yu Y, Chen X, Wei X, Zhang X (2017). Single continuous near-infrared laser-triggered photodynamic and photothermal ablation of antibiotic-resistant bacteria using effective targeted copper sulfide nanoclusters. ACS Appl Mater Interfaces.

[24] Feng Y, Liu L, Zhang J, Aslan H, Dong M (2017). Photoactive antimicrobial nanomaterials. J Mater Chem B.

[25] Ji H, Dong K, Yan Z, Ding C, Chen Z, Ren J (2016). Bacterial hyaluronidase self-triggered prodrug release for chemo-photothermal synergistic treatment of bacterial infection. Small.

[26] Karim MN, Singh M, Weerathunge P, Bian P, Zheng R, Dekiwadia C (2018). Visible-light-triggered reactive-oxygen-species-mediated antibacterial activity of peroxidase-mimic CuO nanorods. ACS Appl Nano Mater.

[27] Meeker DG, Jenkins SV, Miller EK, Beenken KE, Loughran AJ, Powless A (2016). Synergistic photothermal and antibiotic killing of biofilm-associated staphylococcus aureus using targeted antibiotic-loaded gold nanoconstructs. ACS Infect Dis.

[28] Pan WY, Huang CC, Lin TT, Hu HY, Lin WC, Li MJ (2016). Synergistic antibacterial effects of localized heat and oxidative stress caused by hydroxyl radicals mediated by graphene/iron oxide-based nanocomposites. Nanomedicine.

[29] Tan L, Li J, Liu X, Cui Z, Yang X, Zhu S (2018). Rapid biofilm eradication on bone implants using red phosphorus and near-infrared light. Adv Mater.

[30] Wang C, Zhang Q, Wang X, Chang H, Zhang S, Tang Y (2017). Dynamic modulation of enzyme activity by near-infrared light. Angew Chem Int Ed Engl.

[31] Yang Y, Ma L, Cheng C, Deng Y, Huang J, Fan X (2018). Nonchemotherapic and robust dual-responsive nanoagents with on-demand bacterial trapping, ablation, and release for efficient wound disinfection. Adv Funct Mater.

[32] Yin M, Li Z, Ju E, Wang Z, Dong K, Ren J (2014). Multifunctional upconverting nanoparticles for near-infrared triggered and synergistic antibacterial resistance therapy. Chem Commun.

[33] Yang YC, He P, Wang YX, Bai HT, Wang S, Xu JF (2017). Supramolecular radical anions triggered by bacteria *in situ* for selective photothermal therapy. Angew Chem Int Ed Engl.

[34] Zhao Y, Dai X, Wei X, Yu Y, Chen X, Zhang X (2018). Near-infrared light-activated thermosensitive liposomes as efficient agents for photothermal and antibiotic synergistic therapy of bacterial biofilm. ACS Appl Mater Interfaces.

[35] Sharma G, Jagtap JM, Parchur AK, Gogineni VR, Ran S, Bergom C (2020). Heritable modifiers of the tumor microenvironment influence nanoparticle uptake, distribution and response to photothermal therapy. Theranostics.

[36] Zhang WY, Cai K, Li XY, Zhang J, Ma ZY, Foda MF (2019). Au hollow nanorods-chimeric peptide nanocarrier for NIR-II photothermal therapy and real-time apoptosis imaging for tumor theranostics. Theranostics.

[37] Li M, Guan YJ, Zhao AD, Ren JS, Qu XG (2017). Using multifunctional peptide conjugated Au nanorods for monitoring β-amyloid aggregation and chemo-photothermal treatment of Alzheimer's disease. Theranostics.

[38] Gao L, Giglio KM, Nelson JL, Sondermann H, Travis AJ (2014). Ferromagnetic nanoparticles with peroxidase-like activity enhance the cleavage of biological macromolecules for biofilm elimination. Nanoscale.

[39] Labas MD, Zalazar CS, Brandi RJ, Cassano AE (2008). Reaction kinetics of bacteria disinfection employing hydrogen peroxide. Biochem Eng J.

[40] Wu J, Wang X, Wang Q, Lou Z, Li S, Zhu Y (2019). Nanomaterials with enzyme-like characteristics (nanozymes): next-generation artificial enzymes (II). Chem Soc Rev.

[41] Sun HJ, Gao N, Dong K, Ren JS, Qu XJ (2014). Graphene quantum dots-band-aids used for wound disinfection. ACS Nano.

[42] Jia X, Ahmad I, Yang R, Wang C (2017). Versatile graphene-based photothermal nanocomposites for effectively capturing and killing bacteria, and for destroying bacterial biofilms. J Mater Chem B.

[43] Wu Q, Wei G, Xu Z, Han J, Xi J, Fan L (2018). Mechanistic insight into the light-irradiated carbon capsules as an antibacterial agent. ACS Appl Mater Interfaces.

[44] Yu X, He D, Zhang X, Zhang H, Song J, Shi D (2019). Surface-adaptive and initiator-loaded graphene as a light-induced generator with free radicals for drug-resistant bacteria eradication. ACS Appl Mater Interfaces.

[45] Cai S, Jia X, Han Q, Yan X, Yang R, Wang C (2017). Porous Pt/Ag nanoparticles with excellent multifunctional enzyme mimic activities and antibacterial effects. Nano Res.

[46] Ge C, Wu R, Chong Y, Fang G, Jiang X, Pan Y (2018). Synthesis of Pt hollow nanodendrites with enhanced peroxidase-like activity against bacterial infections: implication for wound healing. Adv Funct Mater.

[47] Tao Y, Lin Y, Huang Z, Ren J, Qu X (2013). Incorporating graphene oxide and gold nanoclusters: a synergistic catalyst with surprisingly high peroxidase-like activity over a broad pH range and its application for cancer cell detection. Adv Mater.

[48] Wang N, Hu B, Chen ML, Wang JH (2015). Polyethylenimine mediated silver nanoparticle-decorated magnetic graphene as a promising photothermal antibacterial agent. Nanotechnology.

[49] Wang Z, Dong K, Liu Z, Zhang Y, Chen Z, Sun H (2017). Activation of biologically relevant levels of reactive oxygen species by Au/g-C_3_N_4_ hybrid nanozyme for bacteria killing and wound disinfection. Biomaterials.

[50] Khlebtsov N, Bogatyrev V, Dykman L, Khlebtsov B, Staroverov S, Shirokov A (2013). Analytical and theranostic applications of gold nanoparticles and multifunctional nanocomposites. Theranostics.

[51] Zhang Y, Liu QY, Ma CB, Wang QQ, Yang MT, Du Y (2020). Point-of-care assay for drunken driving with Pd@Pt core-shell nanoparticles-decorated ploy (vinyl alcohol) aerogel assisted by portable pressure meter. Theranostics.

[52] Jeong CJ, Sharker SM, In I, Park SY (2015). Iron oxide@PEDOT-based recyclable photothermal nanoparticles with poly(vinylpyrrolidone) sulfobetaines for rapid and effective antibacterial activity. ACS Appl Mater Interfaces.

[53] Jin Y, Deng J, Yu J, Yang C, Tong M, Hou Y (2015). Fe_5_C_2_ nanoparticles: a reusable bactericidal material with photothermal effects under near-infrared irradiation. J Mater Chem B.

[54] Yu N, Cai T, Sun Y, Jiang C, Xiong H, Li Y (2018). A novel antibacterial agent based on AgNPs and Fe_3_O_4_ loaded chitin microspheres with peroxidase-like activity for synergistic antibacterial activity and wound-healing. Int J Pharm.

[55] Yu S, Li G, Liu R, Ma D, Xue W (2018). Dendritic Fe_3_O_4_@poly(dopamine)@PAMAM nanocomposite as controllable NO-releasing material: a synergistic photothermal and NO antibacterial study. Adv Funct Mater.

[56] Zhang C, Hu DF, Xu JW, Ma MQ, Xing H, Yao K (2018). Polyphenol-assisted exfoliation of transition metal dichalcogenides into nanosheets as photothermal nanocarriers for enhanced antibiofilm activity. ACS Nano.

[57] Zhang W, Shi S, Wang Y, Yu S, Zhu W, Zhang X (2016). Versatile molybdenum disulfide based antibacterial composites for *in vitro* enhanced sterilization and *in vivo* focal infection therapy. Nanoscale.

[58] Misra A, Castillo IF, Müller DP, González C, Eyssautier-Chuine S, Ziegler A (2018). Polyoxometalate-ionic liquids (POM-ILs) as anticorrosion and antibacterial coatings for natural stones. Angew Chem Int Ed Engl.

[59] Yong Y, Zhang C, Gu Z, Du J, Guo Z, Dong X (2017). Polyoxometalate-based radiosensitization platform for treating hypoxic tumors by attenuating radioresistance and enhancing radiation response. ACS Nano.

[60] Yong Y, Zhou L, Zhang S, Yan L, Gu Z, Zhang G (2016). Gadolinium polytungstate nanoclusters: a new theranostic with ultrasmall size and versatile properties for dual-modal MR/CT imaging and photothermal therapy/radiotherapy of cancer. NPG Asia Mater.

[61] Zhang C, Bu WB, Ni DL, Zuo CJ, Cheng C, Li Q (2016). A polyoxometalate cluster paradigm with self-adaptive electronic structure for acidity/reducibility-specific photothermal conversion. J Am Chem Soc.

[62] Yang Z, Fan WP, Tang W, Shen ZY, Dai YL, Song JB (2018). Near-infrared semiconducting polymer brush and pH/GSH-responsive polyoxometalate cluster hybrid platform for enhanced tumor-specific phototheranostics. Angew Chem Int Ed Engl.

