# Supplementary material for: Bacteria responsive polyoxometalates nanocluster strategy to regulate biofilm microenvironments for enhanced synergetic antibiofilm activity and wound healing
Source: Theranostics. 2020 Aug 8;10(22):10031–45. doi: 10.7150/thno.49008 (PMC7481423; doi:10.7150/thno.49008)
Supplement: Supplementary file 1 — Supplementary figures. [file thnov10p10031s1.pdf]

Supporting Information

Bacteria responsive polyoxometalates nanocluster strategy to regulate biofilm microenvironments for enhanced synergetic antibiofilm activity and wound healing

Yuetong Zhang[1#], Yang Pi[1#], Yusheng Hua[1], Jiani Xie[2], Chengyan Wang[2], Kun Guo[3], Zhigang Zhao[1], Yuan Yong[1]*

1. College of Chemistry and Environment Protection Engineering, Southwest Minzu University, Chengdu 610041, China.

2. CAS Key Laboratory for Biomedical Effects of Nanomaterials and Nanosafety, Institute of High Energy Physics, Chinese Academy of Sciences, Beijing100040, China.

3. College of Pharmacy, Southwest Minzu University, Chengdu 610041, China.

[#] These authors contributed equally.

Corresponding Authors:

*E-mail: yongy1816@163.com

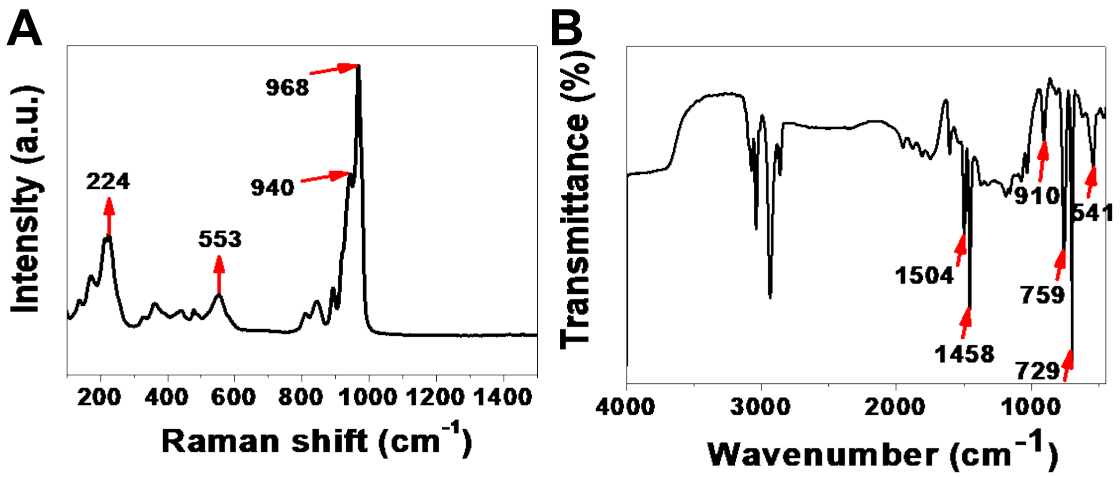

Figure S1. (A-B) Raman and FT-IR spectra of $GdW_{10}O_{36}$ NCs.

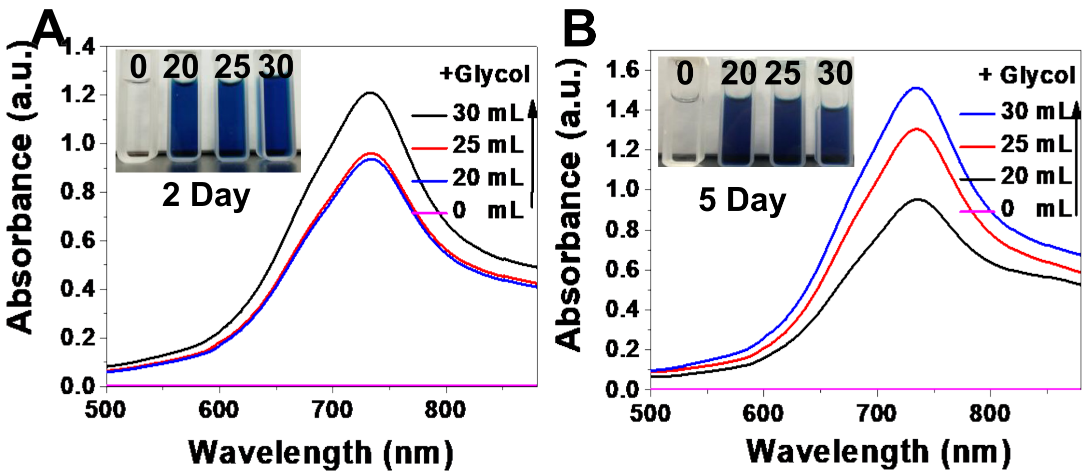

Figure S2. (A-B) The UV-vis spectra and corresponding photographs of various concentrations of ethylene glycol dispersed in deionized water before and after incubation with $GdW_{10}O_{36}$ NCs (10 mg) as a function of time, respectively.

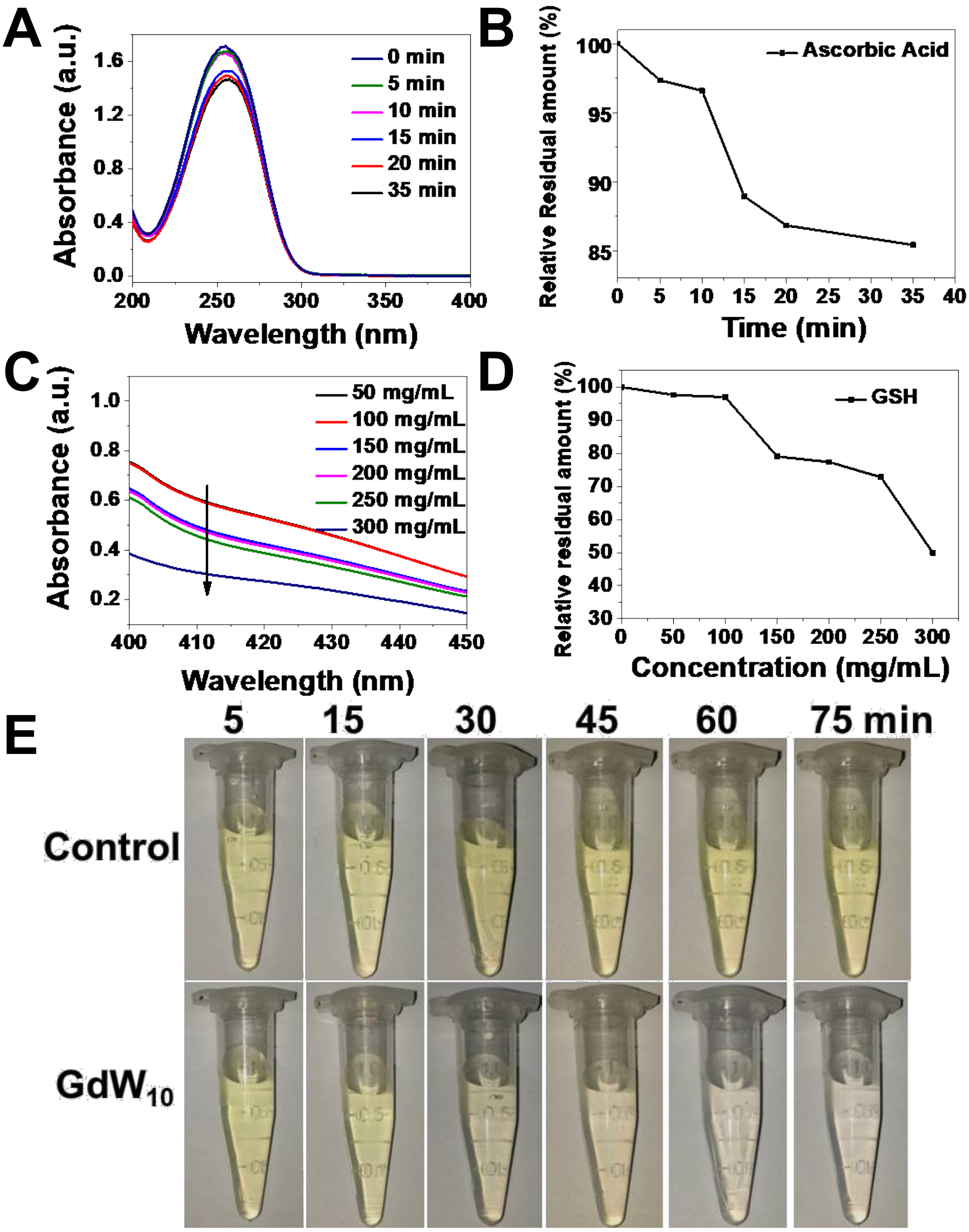

Figure S3. (A-B) The UV-vis spectra and relative residual amount of ascorbic acid before and after incubation with $GdW_{10}O_{36}$ NCs (1.5 mg/mL) as a function of time. (C-D) The UV-vis spectra and relative residual amount of glutathione after incubation with various concentrations of $GdW_{10}O_{36}$ NCs. (E) Photographs for the color change before and after treatment GSH at different time points, determined by the Ellman's assay. The concentration of $GdW_{10}O_{36}$ NCs and GSH was 100 μg/mL and 50 μM. GSH in the absence of $GdW_{10}O_{36}$ NCs was used as control.

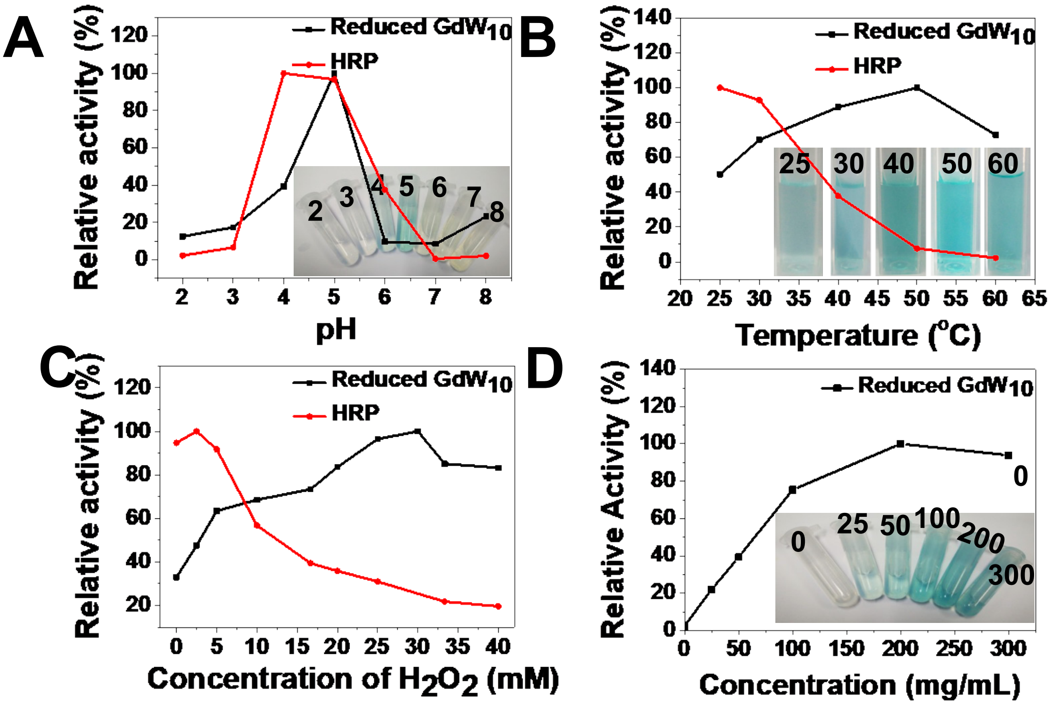

Figure S4. (A) pH-and (B) Temperature-dependent activities with TMB (1 mM), H_2O_2 (33 mM) and with reduced $GdW_{10}O_{36}$ NCs (33 μg/mL).Reduced $GdW_{10}O_{36}$ NCs and HRP show an optimal pH of 4.0–5.0 and optimal temperature around 40–50 °C. (C) H_2O_2 concentration-dependent peroxidase-like activity with reduced $GdW_{10}O_{36}$NCs (33 μg/mL) and TMB (1 mM). Reduced $GdW_{10}O_{36}$ NCs require a higher H_2O_2 concentration than HRP to reach maximal peroxidase activity. (D) Reduced $GdW_{10}O_{36}$ NCs-dependent peroxidase-like activity with TMB (1 mM) and H_2O_2 (33 mM). The maximum point in each curve (a–d) was set as 100 %. Inset shows the photographs of the reaction system.

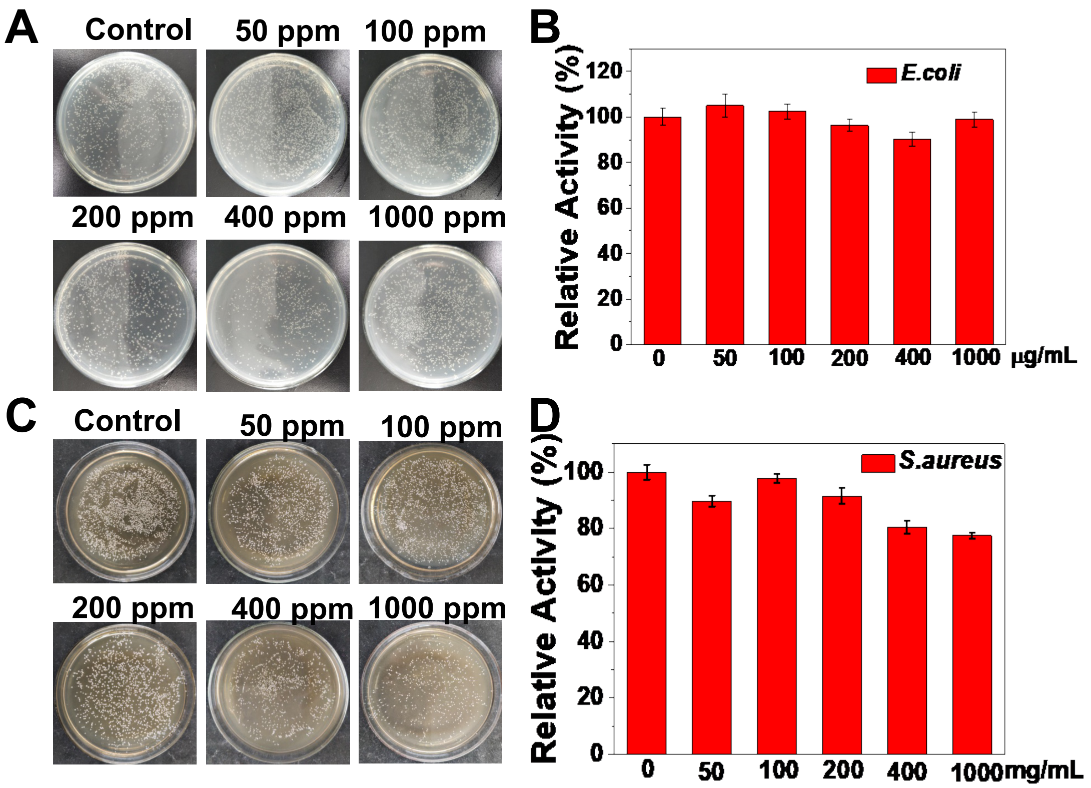

Figure S5. (A, C) Photographs and (B, D) survival rates of *E.coli* and *S.aureus* treated with various concentrations of reduced $GdW_{10}O_{36}$ NCs. The colony-forming units counting method was applied to evaluate the actual antibacterial effects of reduced $GdW_{10}O_{36}$ NCs. The data indicate the means and SD from three parallel experiments.

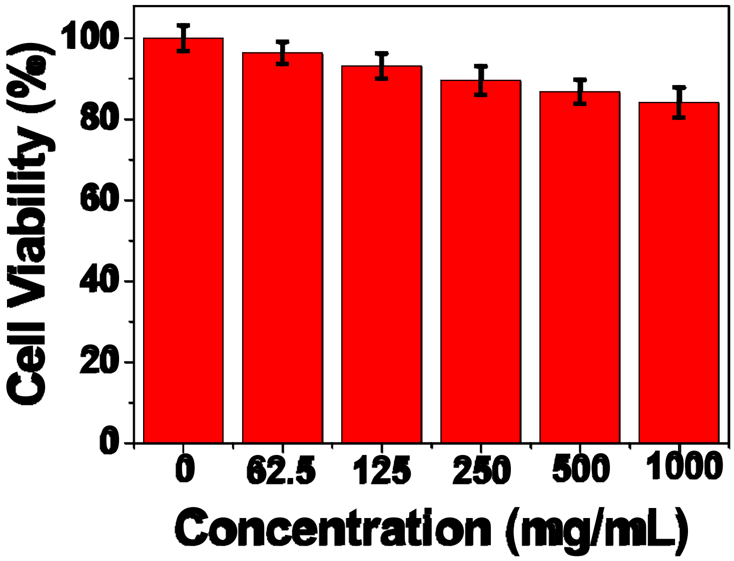

Figure S6. Cell viability assays on 4T1 cells incubated with reduced $GdW_{10}O_{36}$ NCs (0, 62.5, 125, 250, 500 and 1000 μg ml^{-1})

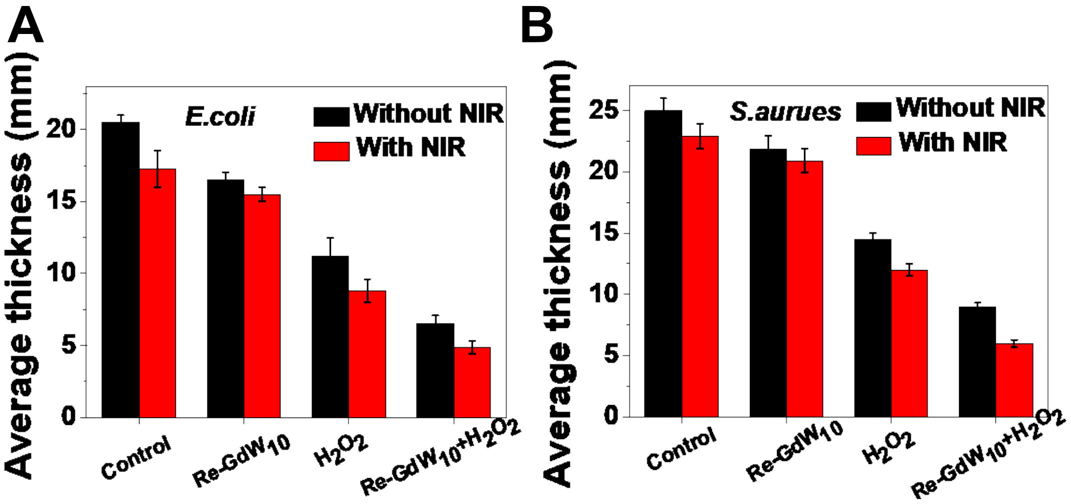

Figure S7. Average thickness of the biofilms shown in (A) *E.coli* and (B) *S.aurues* bacteria as calculated by the COMASTAT software.
